# A Novel, Noncanonical BMP Pathway Modulates Synapse Maturation at the *Drosophila* Neuromuscular Junction

**DOI:** 10.1371/journal.pgen.1005810

**Published:** 2016-01-27

**Authors:** Mikolaj J. Sulkowski, Tae Hee Han, Carolyn Ott, Qi Wang, Esther M. Verheyen, Jennifer Lippincott-Schwartz, Mihaela Serpe

**Affiliations:** 1 Program in Cellular Regulation and Metabolism, Eunice Kennedy Shriver National Institute of Child Health and Human Development (NICHD), National Institutes of Health (NIH), Bethesda, Maryland, United States of America; 2 Cellular Biology and Metabolism Program, Eunice Kennedy Shriver National Institute of Child Health and Human Development (NICHD), National Institutes of Health (NIH), Bethesda, Maryland, United States of America; 3 Department of Molecular Biology and Biochemistry, Simon Fraser University, Burnaby, British Columbia, Canada; Baylor College of Medicine, UNITED STATES

## Abstract

At the *Drosophila* NMJ, BMP signaling is critical for synapse growth and homeostasis. Signaling by the BMP7 homolog, Gbb, in motor neurons triggers a canonical pathway—which modulates transcription of BMP target genes, and a noncanonical pathway—which connects local BMP/BMP receptor complexes with the cytoskeleton. Here we describe a novel noncanonical BMP pathway characterized by the accumulation of the pathway effector, the phosphorylated Smad (pMad), at synaptic sites. Using genetic epistasis, histology, super resolution microscopy, and electrophysiology approaches we demonstrate that this novel pathway is genetically distinguishable from all other known BMP signaling cascades. This novel pathway does not require Gbb, but depends on presynaptic BMP receptors and specific postsynaptic glutamate receptor subtypes, the type-A receptors. Synaptic pMad is coordinated to BMP’s role in the transcriptional control of target genes by shared pathway components, but it has no role in the regulation of NMJ growth. Instead, selective disruption of presynaptic pMad accumulation reduces the postsynaptic levels of type-A receptors, revealing a positive feedback loop which appears to function to stabilize active type-A receptors at synaptic sites. Thus, BMP pathway may monitor synapse activity then function to adjust synapse growth and maturation during development.

## Author Summary

Synaptic activity and synapse development are intimately linked, but our understanding of the coupling mechanisms remains limited. Anterograde and retrograde signals together with trans-synaptic complexes enable intercellular communications. How synapse activity status is monitored and relayed across the synaptic cleft remains poorly understood. The *Drosophila* NMJ is a very powerful genetic system to study synapse development. BMP signaling modulates NMJ growth via a canonical, Smad-dependent pathway, but also synapse stability, via a noncanonical, Smad-independent pathway. Here we describe a novel, noncanonical BMP pathway, which is genetically distinguishable from all other known BMP pathways. This pathway does not contribute to NMJ growth and instead influences synapse formation and maturation in an activity-dependent manner. Specifically, phosphorylated Smad (pMad in flies) accumulates at active zone in response to active postsynaptic type-A glutamate receptors, a specific receptor subtype. In turn, synaptic pMad functions to promote the recruitment of type-A receptors at synaptic sites. This positive feedback loop provides a molecular switch controlling which flavor of glutamate receptors will be stabilized at synaptic locations as a function of synapse status. Since BMP signaling also controls NMJ growth and stability, BMP pathway offers an exquisite means to monitor the status of synapse activity and coordinate NMJ growth with synapse maturation and stabilization.

## Introduction

Bone morphogenetic proteins (BMPs) modulate a wide variety of cellular processes via canonical and noncanonical signaling pathways [1–3]. BMP signaling is initiated when extracellular dimeric BMP ligands bind to a heterotetrameric complex of Ser/Thr kinases, known as type I and type II BMP receptors (BMPR). Following ligand binding, the type II receptor phosphorylates and activates the type I receptor, which in turn phosphorylates the intracellular R-Smad transducer (Smad1, 5 or 8 in vertebrates, and Mad in *Drosophila*) [4,5]. Phosphorylated Smads (pSmads) associate with Co-Smads, and translocate into the nucleus where, in conjunction with other transcription factors, they regulate expression of target genes. Activated BMPRs can also signal independently of Smads through noncanonical pathways, which include various types of mitogen-activated protein kinase (MAPK), LIM (Lin-11/Isl-1/Mec-3 gene products) kinase, phosphatidylinositol 3-kinase/Akt (PI3K/Akt), and Rho-like small GTPases [1,6,7]. Intriguingly, pSmads also accumulate at the cell membrane in at least two instances: (a) at tight junctions during neural tube closure [8], and (b) at the *Drosophila* neuromuscular junction [9,10]. If Smads are not involved in noncanonical BMP pathways and pSmads translocate to the nucleus in response to canonical BMP signaling, then how do pSmads accumulate at membrane locations and why? During neural tube closure, pSmad1/5/8 binds to apical polarity complexes and mediates stabilization of BMP/BMPR complexes at tight junctions [8]; prolonged BMP blockade disrupts the tight junctions and affects epithelial organization [11]. Local pMad accumulation at the fly NMJ requires specific glutamate receptor subtypes [12], but the nature and biological relevance of the pMad-positive puncta remain obscure.

At the *Drosophila* NMJ, BMP signaling controls NMJ growth and promotes synapse homeostasis [13–17]. In the absence of BMP signaling, individual synapses form but the NMJs remain small, with fewer boutons, and exhibit numerous structural and functional defects. Ultrastructural studies indicate that BMP pathway mutants have enlarged active zones, with frequent detachments between the pre- and postsynaptic membranes [14–16]. These mutant NMJs have significantly reduced evoked potentials and lack the ability to induce homeostatic compensatory responses. It is generally thought that BMP signaling fulfills these functions via canonical and noncanonical pathways triggered by Glass-bottom boat (Gbb), a BMP7 homolog, which binds to presynaptic BMPRII, Wishful thinking (Wit), and BMPRIs, Thickveins (Tkv) and Saxophone (Sax). The canonical pathway activates presynaptic transcriptional programs with distinct roles in the structural and functional development of the NMJ [18,19]. For example, the BMP pathway effector Trio, a Rac GEF, can rescue the NMJ growth in BMP pathway mutants, but does not influence synapse physiology, whereas Target of Wit (Twit) can partially restore the mini frequency in *wit* mutants but has no effect on NMJ growth. Besides the canonical BMP signaling, Gbb and the BMP type II receptor Wit signal through the effector protein LIMK1 to regulate synapse stability and addition of new boutons with increased synaptic activity [20,21]. LIMK1 is not required for Mad-mediated NMJ growth; instead, LIMK1 regulates the presynaptic actin dynamics partly by controlling the activity of the actin depolymerizing protein Cofilin. BMP signaling is perturbed in mutants that affect endocytosis, endosomal sorting and retrograde transport, which may disrupt the proper subcellular distribution and transport of BMP/BMPRs signaling complexes to the motor neuron soma [10,22,23]. During development, an early and transient BMP signal is both necessary and sufficient for NMJ growth and activity-dependent synaptic plasticity, whereas the control of NMJ function starts early, during late embryonic stages, and requires continuous BMP signaling throughout development [24].

We have recently discovered that pMad accumulates at synaptic terminals in response to specific glutamate receptor subtypes, the type-A receptors [12]. The fly NMJ ionotropic glutamate receptors (iGluRs) are heterotetrameric complexes composed of three essential subunits–GluRIIC, GluRIID and GluRIIE–and either GluRIIA (type-A receptors) or GluRIIB (type-B) [25–29]. The two receptors have identical single-channel conductances, but type-B receptors desensitize nearly ten times faster that type-A and have reduced quantal size (the postsynaptic response to the fusion of single synaptic vesicles) (reviewed in [30]). GluRIIA and GluRIIB are similarly abundant at the larval NMJ, but they utilize distinct mechanisms for targeting and stabilization at synaptic sites. Furthermore, GluRIIA competes with GluRIIB for the limiting essential subunits, GluRIIC, -D and -E, so an increase in synaptic GluRIIA induces a decrease in GluRIIB [27]. Studies on mutant GluRIIA variants indicate that channel properties also influence trafficking and synaptic distribution of GluRIIA [26,31]. GluRIIA is both necessary and sufficient for the experience-dependent strengthening and growth of the NMJ [32]. pMad accumulates at synaptic terminals at the onset of synaptogenesis and mirrors postsynaptic GluRIIA throughout the NMJ development [12].

Here we examine the regulation and function of synaptic pMad during NMJ development. We found that synaptic pMad is generated in the presynaptic compartment via a novel BMP signaling pathway that is genetically distinguishable from the canonical BMP signaling and the Wit/LIMK1 noncanonical pathway. This novel pathway does not require Gbb, but depends on presynaptic Wit and Sax and postsynaptic GluRIIA receptors. Using genetic epistasis experiments, we demonstrate that synaptic pMad has no role in the regulation of NMJ growth. Instead, selective disruption of presynaptic pMad accumulation reduced postsynaptic GluRIIA levels, revealing a positive feedback loop which appears to function to stabilize active type-A receptors at synaptic sites.

## Results

**Fig 1:**
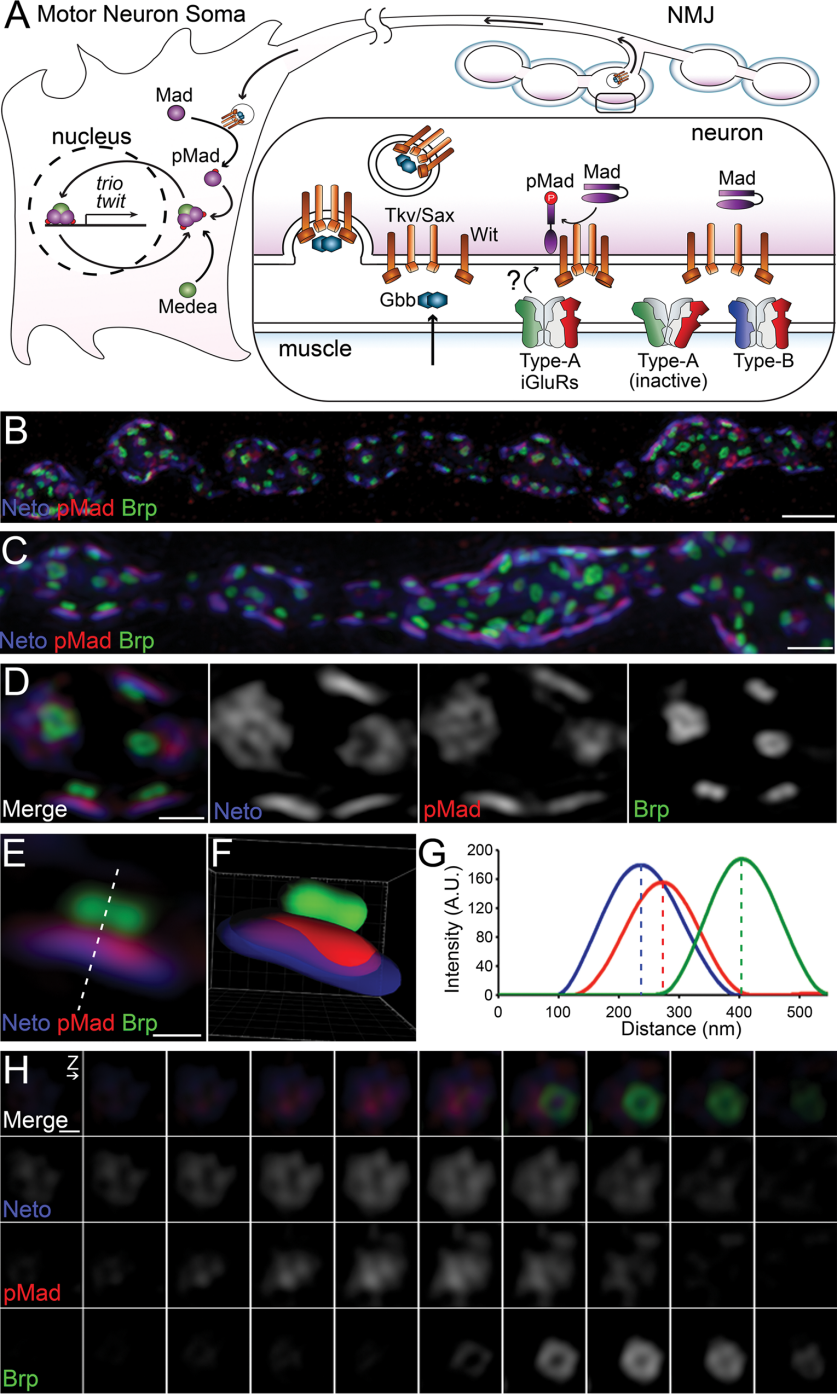
Synaptic pMad localizes at the active zone. (A) Diagram of BMP signaling complexes that control the accumulation of nuclear and synaptic pMad. Extracellular BMPs bind to a complex composed of Type I and Type II BMP receptors. The BMP/BMPR complexes are endocytosed and transported to the neuron soma, where they phosphorylate Mad and allow for translocation and accumulation of pMad in the motor neuron nuclei. Synaptic pMad mirrors the active postsynaptic GluRIIA and likely reflects local accumulation of BMP/BMPR complexes. (B-D) 3D-SIM images of NMJ12 boutons from third instar larvae labeled for Brp (green), pMad (red), and Neto (blue). SIM z stack maximum projections are shown in (A–B) and a single z plane is shown in (C). See also S1 and S2 Movies. (E) High magnification view of a single synapse profile (from panel C). The line indicates the position used for the linescan plotted in panel F. (F) Side view of a surface rendered volume of the synapse shown in panel D. (G) Intensity profile of Neto, pMad and Brp signal along the line drawn in panel E. Linescans like this were performed across many synapses to measure the distance of pMad and Neto from Brp. (H) High magnification view of a z series through a single synapse imaged *en face*. The z interval was 110nm. Both merged and individual channels are shown. See also S3 Movie. Scale bars: 2 μm (B), 1 μm (C), 500 nm (C), 200 nm (E and H).

We have previously demonstrated that synaptic but not nuclear pMad levels in the motor neurons correlate with active postsynaptic type-A receptors at the *Drosophila* NMJ [12]. Genetic manipulations of GluRIIA levels and activity status induce proportional changes in synaptic pMad levels, but have no effect on nuclear pMad accumulation. Since BMP signaling is short lived [33,34], synaptic pMad likely marks active BMP/BMPR complexes present at synaptic terminals (Fig 1A). This suggests that the presynaptic compartment contains two distinct pools of pMad: (1) a nuclear pool, which controls the transcriptional response to BMP retrograde signaling and (2) a local pool, regulated by postsynaptic Neto/type-A glutamate receptors.

Unlike nuclear pMad, which uniformly labels the motor neuron nuclei, local pMad decorates the synaptic boutons as distinct puncta, which accumulate at synaptic sites. To examine the subcellular localization of pMad signals within the synaptic regions we analyzed neuromuscular synapses stained for pre- and postsynaptic components using 3D structured illumination microscopy (3D-SIM) (Fig 1B–1H). At the *Drosophila* NMJ synapses, the sites of neurotransmitter release are marked by presynaptic specializations called T-bars, where Bruchpilot (Brp), the fly homolog of the vertebrate active zone protein ELKS, accumulates [35]. The anti-Brp monoclonal antibody Nc82 recognizes an epitope on the outer diameter of the T-bars and produces a ring-shaped signal when examined by STED [36] or 3D-SIM (Fig 1B–1D). Using a line plot across a single z slice and measuring the distance between the local maxima, we estimated the Brp diameter to be 139 nm (SE± 8; n = 11). Opposite to the T-bars, the postsynaptic densities (PSDs) comprise a myriad of proteins that concentrate and stabilize the iGluRs. Clustering of iGluRs at synaptic sites requires Neto (Neuropillin and Tolloid-like), an essential auxiliary subunit that colocalizes with the iGluRs at the PSDs and modulates their distribution and function [37–40]. We imaged synapses immunostained for Neto using an antibody against the extracellular CUB1 domain of Neto, which resides within the 20 nm synaptic cleft. The Neto-positive signals distributed into thin discs of irregular shapes, juxtaposing each Brp-positive T-bar (Fig 1D, S1 and S2 Movies). The size and position of Neto domains are consistent with Neto labeling each iGluR/PSD field within the synaptic cleft (see below). The optimal lateral and axial resolution of 3D-SIM is ~100 nm and ~250 nm, respectively. To measure the distance between the T-bar and the synaptic cleft, we plotted the fluorescence intensity of lines drawn across xy profiles of synapses (as shown in Fig 1E). The distance between the Brp and Neto peaks was 114 nm (SE ± 9; n = 28). Interestingly, 3D-SIM revealed that like Neto, pMad is also distributed into discs of irregular shapes (Fig 1D). Both surface rendering and profile linescans show that the fluorescence intensity of Neto and pMad partly overlap (Fig 1D–1H and S3 Movie). We clearly resolve the distance between Brp and pMad (106 nm, SE ± 7; n = 28). Since the Neto domains mark the active zone synaptic cleft, our data show that pMad localizes at the active zone in close proximity to the presynaptic membrane.

### Local pMad accumulates in the presynaptic compartment

Previous light microscopy studies argued that pMad localizes to both pre- and postsynaptic compartments [9,41]. However, loss of *wit* effectively eliminates the synaptic pMad signals [12,42]. As Wit is known to function and to be predominantly expressed in the motor neurons, it was inferred that synaptic pMad was presynaptic. Since 3D-SIM resolution is not sufficient to address this issue, we set up a tissue specific rescue experiment using a *mad* deficiency chromosome and a strong hypomorphic *mad* allele, *mad*^*12*^, which produces a truncated Mad without the last 39 residues, including the site of BMP-dependent phosphorylation [43].

**Fig 2:**
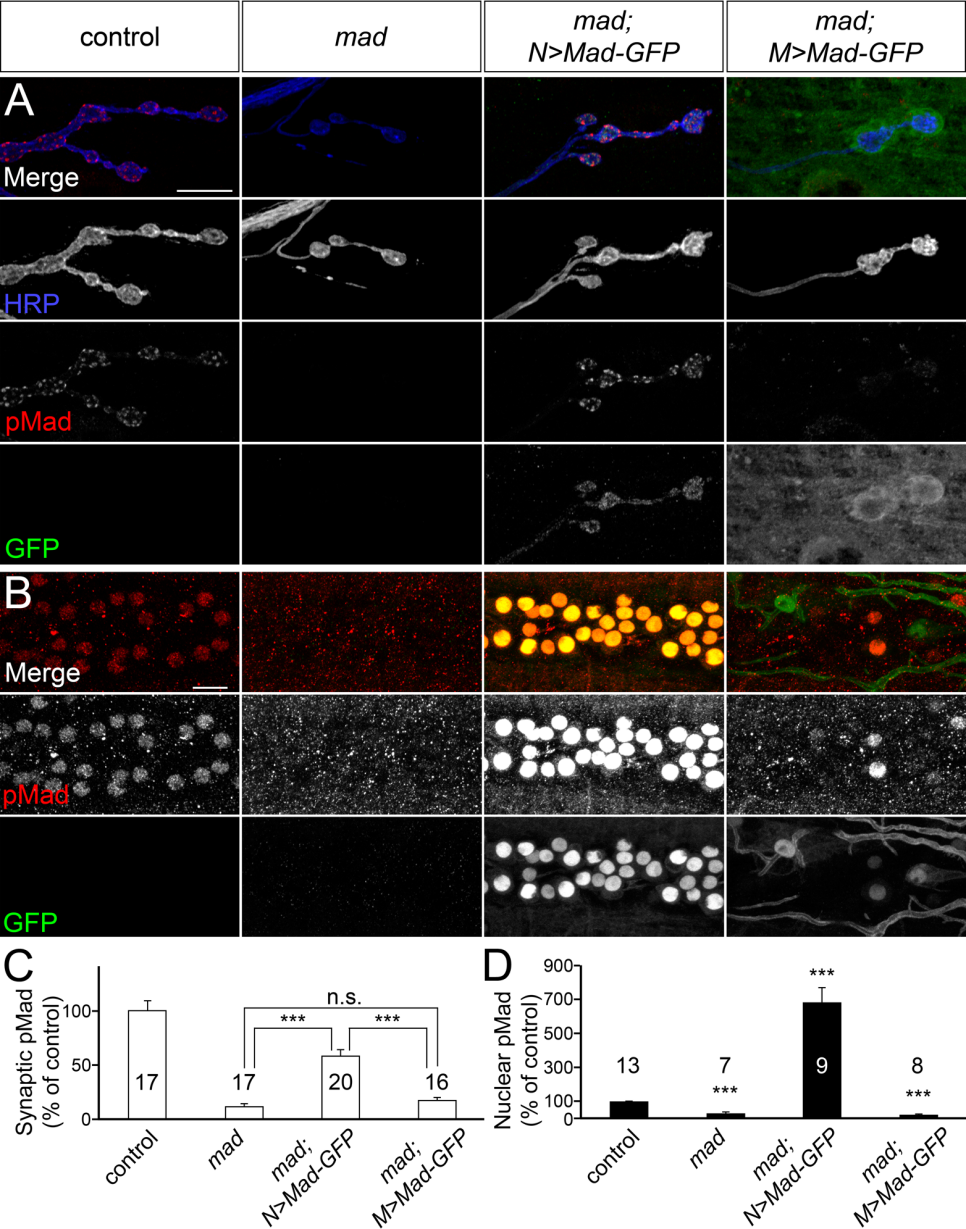
Pre- but not postsynaptic expression of Mad-GFP restores synaptic pMad in *mad* mutants. (A-D) Confocal images of NMJ4 boutons (A) or ventral ganglia (B) (quantified in C-D) from third instar larvae immunostained for pMad (red), GFP (green), and HRP (blue). (A) Lack of synaptic pMad at *mad* null mutants is restored by expression of Mad-GFP in motor neurons (*mad; N>Mad-GFP*). Muscle expression of Mad-GFP (*mad; M>Mad-GFP*) does not rescue synaptic pMad, even though Mad-GFP accumulates around synaptic boutons. (B) Expression of Mad-GFP in motor neurons of *mad* mutants leads to elevated nuclear pMad levels. Muscle expression of Mad-GFP does not restore nuclear pMad in *mad* mutants, except for a small subset of neurons expressing Mad-GFP, which were excluded from quantification. Genotypes: control (*w*^*1118*^), *mad* (*mad*^*12/Df*^), *mad; N>Mad-GFP (380-Gal4/+; mad*^*12/Df*^*; UAS-Mad-GFP/+*), *mad; M>Mad-GFP* (*mad*^*12/Df*^*; 24B-Gal4/UAS-Mad-GFP)*. Error bars indicate SEM. ***; p<0.001. Scale bars: 10 μm.

As expected, both synaptic and nuclear pMad signals were largely absent from these *mad* mutants (*mad*^*12/Df*^), which die as translucent third instar larvae, with almost no fat body (Fig 2). Expression of Mad-GFP in pre-synaptic motor neurons of *mad* mutants did not rescue the adult viability but was sufficient to restore both nuclear and synaptic pMad levels during larval stages. In fact, the nuclear pMad levels were greatly increased (by 685 ± 88%, n = 9) in the motor neuron nuclei of rescued animals as compared with wild-type controls. The synaptic pMad levels increased from 13 ± 2% at *mad* mutant NMJs to 58 ± 6% (p<0.0001, n = 20) in the rescued larvae relative to controls. In contrast, expressing Mad-GFP in the postsynaptic muscle did not restore synaptic pMad. These larvae had severely enlarged boutons, marked by GFP-positive signals, indicating that Mad-GFP accumulates at postsynaptic locations, but their synaptic pMad levels remained similar to those measured at *mad* mutant NMJs. Nuclear pMad was also largely absent from these rescued larvae, except for a small subset of neurons which express the *24B-Gal4* line used here. The fact that Mad expressed in motor neurons, but not in muscle, restored the synaptic pMad at *mad* mutant NMJs unambiguously demonstrates that synaptic pMad resides primarily in the presynaptic compartment.

### Synaptic pMad is not required for NMJ overgrowth

**Fig 3:**
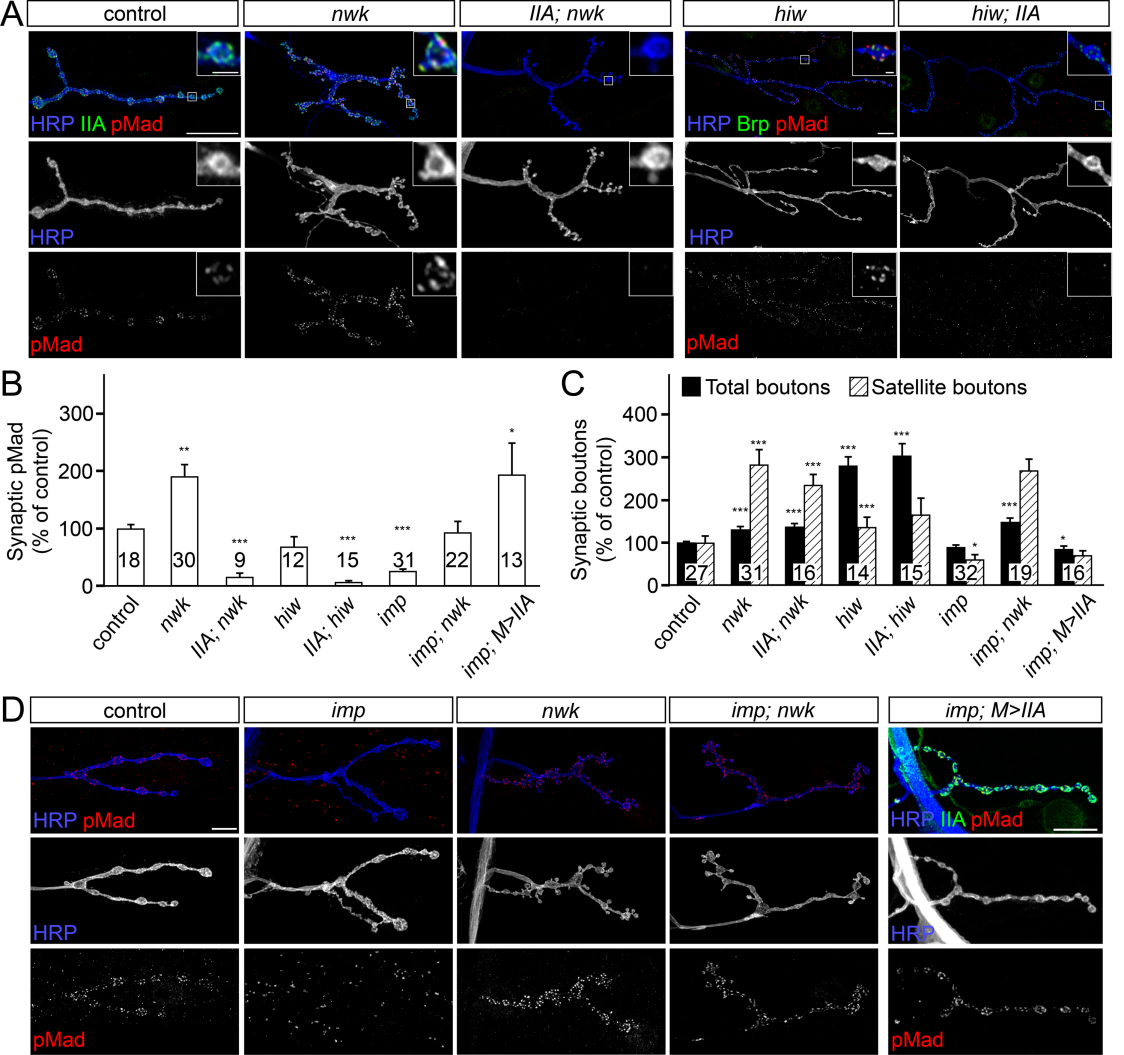
Synaptic pMad is dispensable for the NMJ overgrowth. (A-D) Confocal images of NMJ4 boutons (A and D) (quantified in B-C) from larvae of indicated genotypes immunostained for pMad (red), HRP (blue), which labels the neuronal surface, and GluRIIA or Brp (green). (A) pMad localizes as discrete puncta at control and *nwk* or *hiw* mutant NMJs, but is absent in *GluRIIA; nwk* and *hiw; GluRIIA* double mutants. Loss of synaptic pMad caused by mutations in *GluRIIA* does not rescue the aberrant morphology of *nwk* mutant NMJs, and not prevent the NMJ overgrowth of *hiw* mutants. (D) Local pMad levels are reduced at *imp* mutant NMJs and elevated in *nwk* mutants. Loss of *nwk* restores the synaptic pMad at *imp* NMJs (*imp; nwk*), but morphology remains similar to *nwk* alone. Muscle overexpression of *GluRIIA* restores the synaptic pMad, but not the NMJ growth to *imp* mutants (*imp; M>IIA*). The number of NMJs examined is indicated in each bar. Genotypes: control (*w*^*1118*^), *nwk* (*nwk*^*1/γ3*^), *IIA; nwk* (*GluRIIA*^*SP16/Df*^*; nwk*^*1/γ3*^), *hiw* (*hiw*^*ND8*^), *hiw; IIA* (*hiw*^*ND8*^*; GluRIIA*^*SP16/Df*^), *imp* (*imp*^*24/70*^), *imp; nwk* (*imp*^*24/70*^; *nwk*^*1/γ3*^), *imp; M>IIA* (*UAS-GluRIIA*/*+*; *imp*^*24/70*^; *24B-Gal4/+)*. Error bars indicate SEM. ***; p<0.001, **; p<0.01, *; p<0.05. Scale bars: 10 μm and 1 μm (details).

Elevated levels of synaptic pMad were previously correlated with synaptic overgrowth, in particular with the presence of supernumerary/satellite boutons observed in many endocytic mutants [44]. For example, synaptic pMad and the number of satellite boutons are elevated in the absence of Nervous wreck (Nwk), an adaptor protein which appears to link Tkv, the type I BMPR, with the endocytic machinery [45]. To test whether synaptic pMad directly influences the formation of satellite boutons we set up a series of genetic epistasis experiments. We found that local pMad signals were completely abolished in *nwk; IIA* double mutants (Fig 3A and 3B). This is consistent with our previous finding that synaptic pMad is absolutely dependent on postsynaptic GluRIIA. However, loss of synaptic pMad in the *nwk; IIA* double mutants did not alleviate the aberrant morphology observed in *nwk* mutants; these NMJs remained overgrown with a high number of satellite boutons, similar to mutations in *nwk* alone (Fig 3A and 3C). Thus, synaptic pMad does not influence the *nwk*-dependent NMJ overgrowth.

*Drosophila* NMJs grow exuberantly and are greatly expanded in the absence of Highwire (Hiw), a conserved E3 ubiquitin ligase that limits synaptic growth [17,46,47]. It has been shown that BMP signaling mutants suppress the excessive NMJ growth of *hiw* mutants [17]. We found that NMJs in *hiw; IIA* double mutants selectively lose synaptic pMad signals, but they remain overgrown, and resemble the *hiw* mutant NMJs (Fig 3A, 3B and 3C). These findings indicate that synaptic pMad is not required for the NMJ overgrowth.

### GluRIIA is sufficient for synaptic accumulation of pMad

Previous studies demonstrate that synaptic pMad signals are selectively lost in *importin-ß11* mutants (*impß11*) but could be restored by excess presynaptic BMPRs or by blocking retrograde transport in the motor neurons [42]. Since Nwk limits the retrograde BMP signaling partly by controlling Tkv turnover [45], we asked whether *nwk* is epistatic to *impß11*. We found that the local pMad levels at *impß11*; *nwk* double mutant NMJs were restored to normal levels (Fig 3B–3D). However, the NMJ morphology of *impß11*;*nwk* double mutants resembled the characteristic *nwk* mutant phenotype, *i*.*e*. overgrown NMJs with numerous satellite boutons. These results indicate that *nwk* is epistatic to *impß11* in controlling the NMJ morphology and growth, but *impß11* and *nwk* appear to influence the local pMad accumulation through distinct pathways.

Intriguingly, overexpression of Mad cannot rescue the loss of synaptic pMad at *impß11* NMJs [42]. How can excess BMPRs restore the pMad signals at *impß11* NMJs while excess Mad cannot? Since synaptic pMad presumably marks local BMP/BMPR active complexes, excess neuronal BMPRs may restore the synaptic BMP/BMPR pool and thus rescue the accumulation of pMad at *impß11* mutant NMJs, whereas in the absence of synaptic BMP/BMPR complexes, Mad could not be phosphorylated locally even when in excess. We have previously shown that GluRIIA muscle expression increases local pMad due to increased synaptic type-A receptors [12]. Interestingly, GluRIIA muscle expression efficiently rescued the pMad levels at *impß11* NMJs, although it did not alleviate their growth defects (Fig 3B–3D). This result has two implications: First, it demonstrates that GluRIIA is sufficient for presynaptic pMad accumulation. Second, since presynaptic BMPRs also restore local pMad at *impß11* NMJs [42], then postsynaptic GluRIIA and presynaptic BMPRs likely function together to trigger pMad accumulation at synaptic terminals. Impß11 presumably limits the retrograde transport of BMP/BMPR complexes and thus further stabilizes local pMad.

Additional endocytic components including Spichthyin, Endophilin, Spinster and Liquid facets, the *Drosophila* homolog of Epsin1, limit the local pMad pool [23,48]. We found that removal of GluRIIA in any of the endocytic mutants tested induced complete loss of synaptic pMad. Thus, GluRIIA is both required and sufficient for the synaptic accumulation of pMad. While genetic manipulations of postsynaptic GluRIIA receptors induced proportional changes in the level of synaptic pMad, such manipulations had no detectable effect on the nuclear pMad [12]. This implies that genetically distinct pathways regulate the nuclear and local pMad pools.

### Nuclear and local pMad accumulation are independently regulated

**Fig 4:**
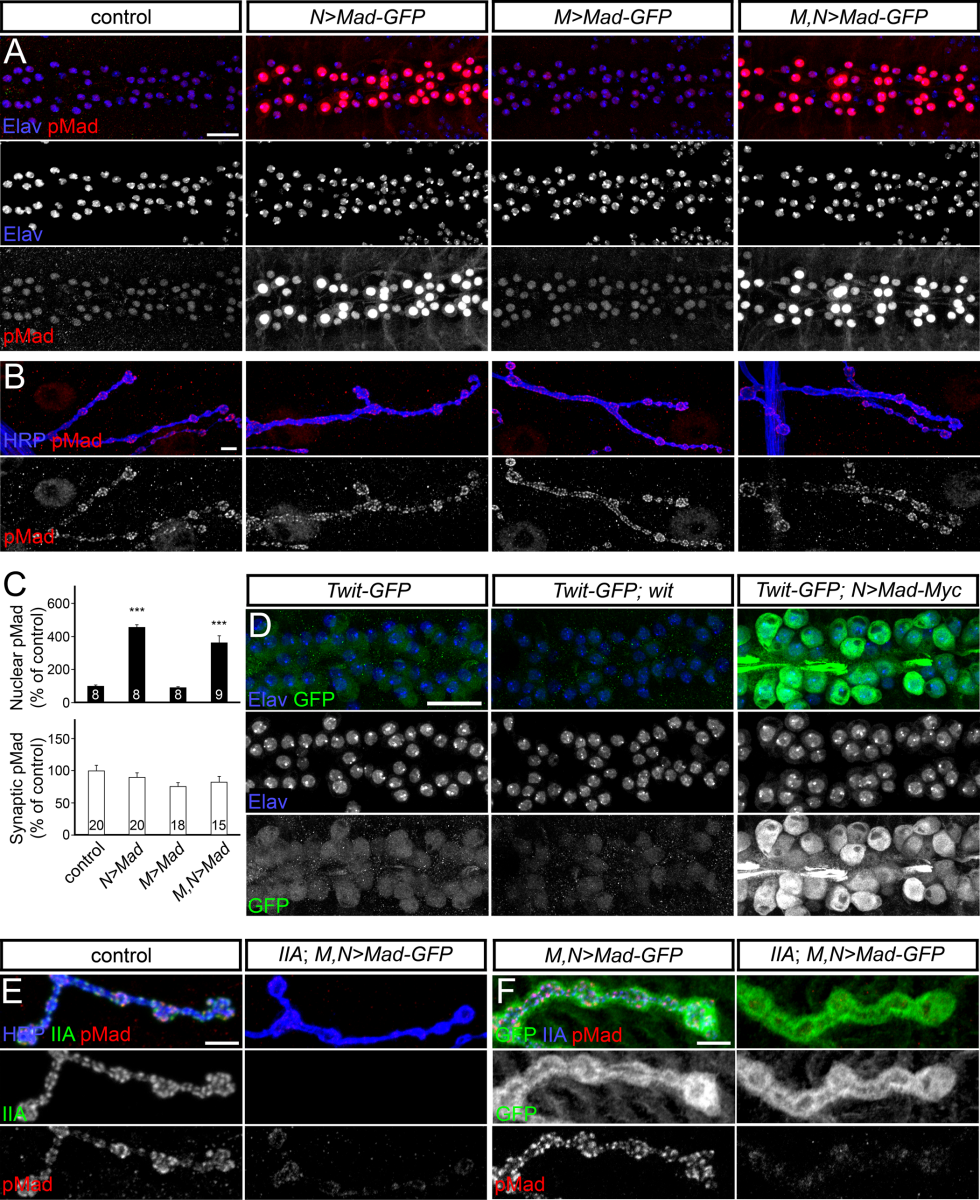
Mad levels do not limit local pMad accumulation. (A) Confocal images of ventral ganglia of third instar larvae of indicated genotypes labeled for pMad (red) and Elav (blue), which marks the motor neuron nuclei. Nuclear pMad is greatly increased when Mad-GFP is expressed in neurons (*N>Mad-GFP*) but not when expressed in muscle (*M>Mad-GFP*). (B) None of these manipulations affect the accumulation of pMad at synaptic sites, as indicated in confocal images of NMJ4 boutons (quantified in (C)). (D) Neuronal expression of Twit-GFP (green), a MiMIC-generated chimera, provides a read-out for the retrograde BMP signaling. Twit-GFP levels are not detectable in *wit* mutants, and are elevated when Mad is overexpressed in motor neurons. (E-F) Confocal images of NMJ4 boutons from larvae of indicated genotypes labeled for pMad, GluRIIA and HRP (E), or GFP (F). Local pMad is lost at *GluRIIA* mutant NMJs even when Mad-GFP is overexpressed and abundantly present at synaptic terminals. Genotypes: control (*w*^*1118*^), *N>Mad-GFP* (*380-Gal4/+; +; UAS-Mad-GFP/+*), *M>Mad-GFP* (*24B-Gal4/UAS-Mad-GFP*), *M*,*N>Mad-GFP* (*elav-Gal4*, *24B-Gal4/UAS-Mad-GFP*), *Twit-GFP* (*Mi(MIC)twit*^*MI06552*^*/+*), *Twit-GFP;wit*^*A12/Df*^ (*Mi(MIC)twit*^*MI06552*^*/+*;*wit*^*A12/Df*^), *Twit-GFP;N>Mad-Myc* (*380-Gal4/+; Mi(MIC)twit*^*MI06552*^*/+; UAS-Mad-Myc*). Error bars indicate SEM. ***; p<0.001. Scale bars: 20 μm (A and D) and 5 μm (B, E and F).

Smad levels and activities are tightly controlled by posttranslational regulation [49,50]. To test whether the levels of Mad are limiting for synaptic pMad accumulation, we examined the effect of excess Mad. Overexpression of Mad-GFP in motor neurons, but not in muscles, produced a dramatic increase of pMad in motor neuron nuclei (Fig 4A). However, the synaptic pMad remained unchanged irrespective of excess Mad in the pre- and/or postsynaptic compartments (Fig 4B and 4C). No change in local pMad was observed for various tagged and non-tagged *Mad* transgenes (see below). These *Mad* transgenes were functional as indicated by their ability to induce increased expression of *twit*, a BMP transcriptional target (Mad-Myc shown in Fig 4D). Twit levels were directly monitored via a MiMIC insertion line, *twit*^*MI06552*^ [51], which generates a Twit-GFP chimera: Twit-GFP was reduced in the absence of *wit*, and was strongly increased when Mad was overexpressed in motor neurons (Fig 4D).

Overexpression of *Mad-GFP* in *GluRIIA* mutants induced high levels of nuclear pMad, but pMad was undetectable at synaptic locations (Fig 4E). Lack of synaptic pMad cannot be explained by a deficit in Mad-GFP axonal trafficking and/or local translation since high levels of Mad-GFP accumulated at synaptic terminals in control and *GluRIIA* mutants (Fig 4F). Instead, our data demonstrate that synaptic pMad is absolutely dependent on postsynaptic GluRIIA receptors and is not limited by the net levels of Mad.

### Gbb is not required for local pMad accumulation

**Fig 5:**
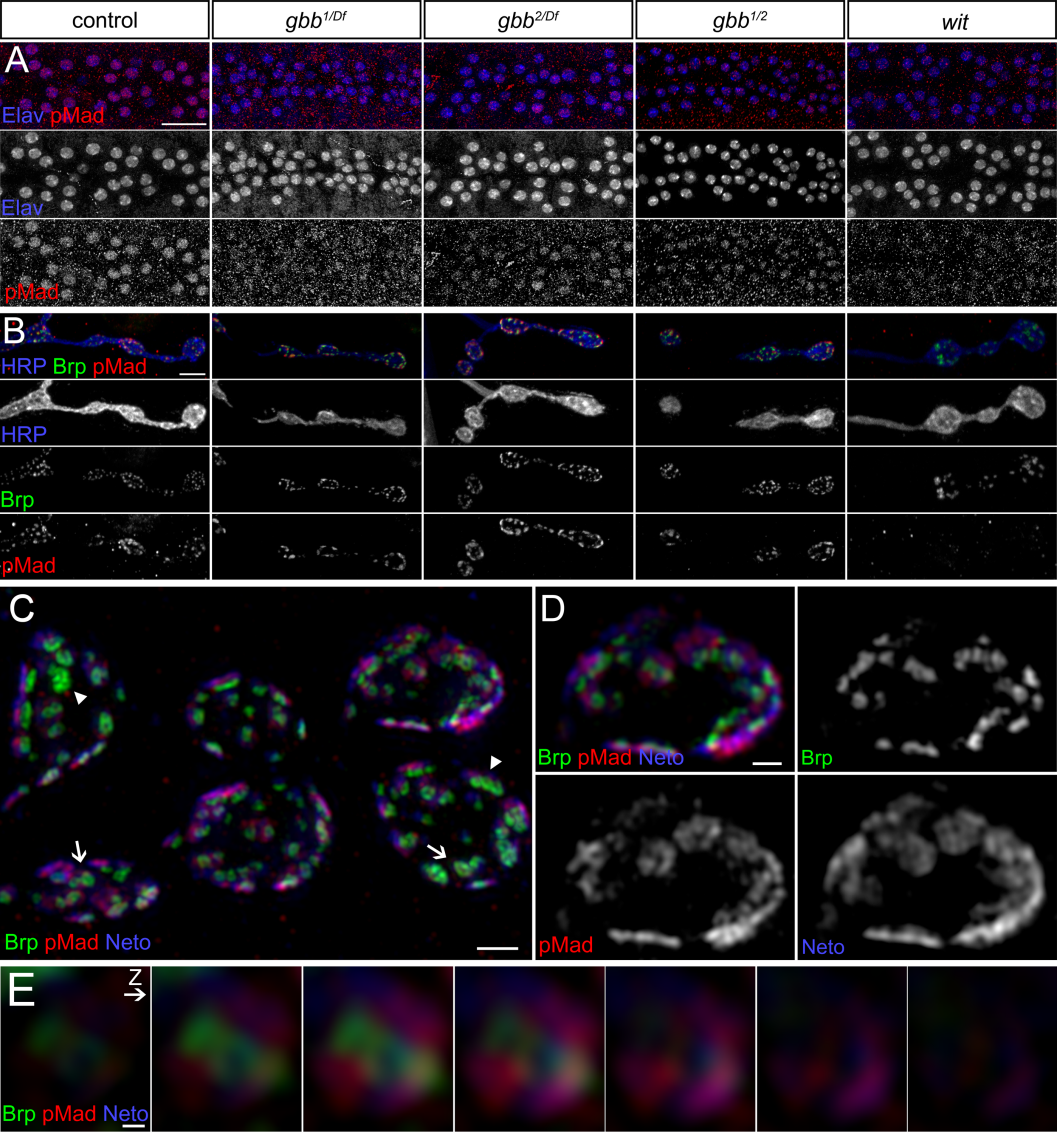
Gbb is not required for local pMad accumulation. (A-B) Confocal images of ventral ganglia (A) and NMJ4 boutons (B) from larvae of indicated genotypes labeled for pMad (red) and Elav (blue) (A), or Brp (green) and HRP (blue) (B). Nuclear pMad is greatly reduced in both *gbb* and *wit* mutants, but synaptic pMad appears normal in all *gbb* null alleles tested. (C) Maximum intensity projection of 3D-SIM images of NMJ12 boutons from third instar *gbb* mutant larvae labeled for Brp (green), pMad (red), and Neto (blue). Arrows, enlarged active zones; arrowheads, multiple T-bars. (D) A single z plane of the top right bouton in panel (C) magnified. See also S4 Movie. SIM z stack maximum projections are shown in (C) and single z plane in (D). (E) High magnification view of a 3D-SIM z series through of an individual *gbb* mutant synapse imaged *en face*. See also S5 Movie. Scale bars: 20 μm (A), 5 μm (B), 1 μm (C), 500 nm (D), 100 nm (E).

While Gbb is absolutely required for the nuclear accumulation of pMad and transcriptional regulation of BMP target genes, we found that Gbb is dispensable for local pMad accumulation (Fig 5A and 5B). In *gbb* mutant animals, the mean pMad level per NMJ was similar in intensity to control animals (*w*^*1118*^) (quantified in S1 Fig). Significant levels of synaptic pMad were observed in animals transheterozygous for different combinations of *gbb* null alleles, ruling out the contribution of the genetic background to this unexpected result. In contrast, synaptic pMad was completely lost at *wit* mutant NMJs (Fig 5B and [12]).

Super resolution imaging of *gbb* mutant boutons showed enlarged active zones (Fig 5C), consistent with ultrastructural defects reported for BMP pathway mutants [14,17]. In particular, a striking feature of BMP mutant synapses is increased accumulation of presynaptic T-bar material, some of it detached from the presynaptic membrane, and the appearance of active zone profiles with two or more T-bars, presumably due to perturbed Brp recruitment [24,32]. Indeed, at *gbb* mutant boutons we found many synapses decorated by multiple or enlarged Brp-positive rings. These enlarged Brp domains juxtaposed large Neto-positive fields that appear to include both enlarged (arrows) and multiple (arrowheads) PSDs (Fig 5D and 5E and S4 and S5 Movies). Nonetheless, pMad was present at all *gbb* mutant active zones—adjacent to Brp and partially overlapping with Neto. These data indicate that Gbb and canonical BMP signaling regulate the assembly and/or maintenance of presynaptic T-bars, likely via BMP transcriptional targets. However, Gbb is not essential for the accumulation of pMad at active zones.

Previous studies have reported an absence of synaptic pMad in *gbb* mutants, or residual pMad signals due to leaky transgenes [24]. We considered whether environmental conditions could account for difference in synaptic pMad levels in our studies versus others, especially since *gbb* has been implicated in regulating nutrient storage and energy homeostasis [52]. It has been found empirically that more *gbb* mutant larvae progress to third instar when raised on a yeast-rich diet. We found that rearing *gbb* mutants exclusively on yeast paste induced a drastic reduction of local pMad (S1 Fig). Furthermore, wild-type animals reared on yeast paste also showed a dramatic reduction of synaptic pMad (up to 5 fold) but normal nuclear pMad. The loss of synaptic pMad could be caused by reduced NMJ activity and limited locomotion as these animals no longer roam for food, or may reflect a response to the nature of food and/or other factors. Nonetheless, our results underscore the importance of rearing conditions when examining BMP signaling at the larval NMJ.

**Fig 6:**
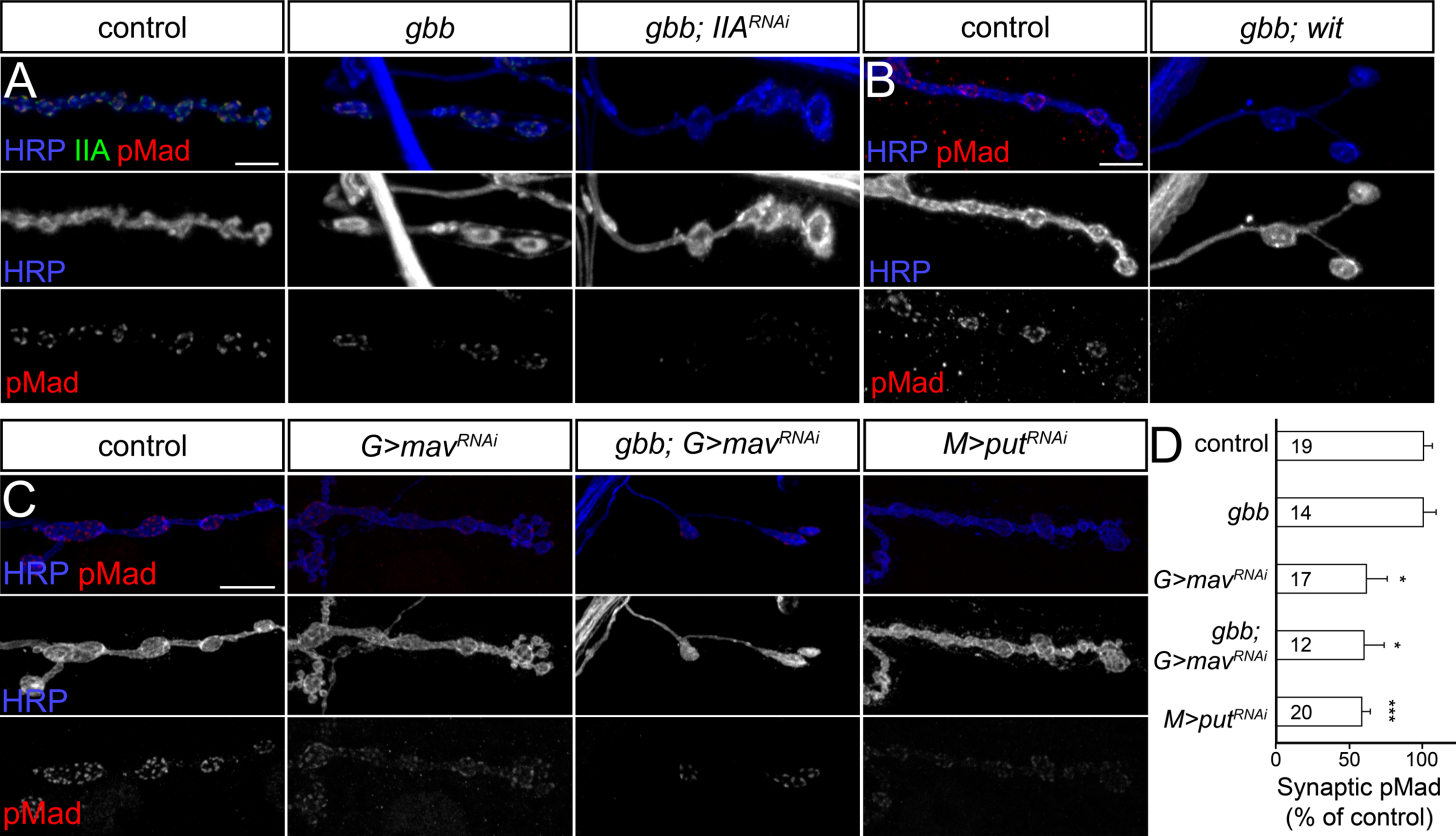
Complex genetic control for synaptic pMad accumulation. (A-C) Confocal images of NMJ4 boutons from larvae of indicated genotypes labeled for pMad (red), HRP (blue) and GluRIIA (green). The accumulation of pMad at *gbb* mutant NMJs is reduced by postsynaptic *GluRIIA* knockdown (A) or loss of Wit (B). Knockdown of Mav in the glia or Put in the striated muscle (C) diminished the synaptic pMad accumulation (quantified in D). Genotypes: control (*w*^*1118*^), *gbb* (*gbb*^*1/2*^), *gbb; IIA*^*RNAi*^ (*gbb*^*1/2*^; *UAS-GluRIIA*^*RNAi*^/*24B-Gal4*), *gbb; wit* (*gbb*^*1/2*^; *wit*^*A12/Df*^), *G>mav*^*RNAi*^ *(repo-Gal4/UAS-mav*^*RNAi*^*)*, *gbb; G>mav*^*RNAi*^ *(gbb*^*1/2*^*; repo-Gal4/UAS-mav*^*RNAi*^, *M>put*^*RNAi*^ *(G14-Gal4/UAS-put*^*RNAi*^*)*. Error bars indicate SEM. ***; p<0.001, *; p<0.05. Scale bars: 5 μm.

Similar to controls, knockdown of *GluRIIA* in the *gbb* mutant background drastically diminished the synaptic pMad levels, indicating that local pMad requires GluRIIA, albeit not Gbb (Fig 6A). Furthermore the local pMad was lost at *gbb; wit* double mutant NMJs (Fig 6B), demonstrating that Wit is absolutely required for the accumulation of synaptic pMad. Together these data show that Gbb, but not Wit or GluRIIA, is dispensable for the synaptic pMad accumulation. Other(s) ligand may promote accumulation of synaptic BMPR complexes that phosphorylate Mad locally.

It was previously shown that glia-derived Maverick (Mav) is secreted in the synaptic cleft and signals to the postsynaptic muscle via the BMPR type II Punt (Put) to modulate NMJ development partly by controlling Gbb expression [41]. Intriguingly, knockdown of Mav in glia also triggered a dramatic reduction of synaptic pMad, raising the possibility that Mav may also signal to the presynaptic neuron to influence pMad accumulation. To test this scenario, we first confirmed that depletion of glia-derived Mav using the available TRiP lines reduced the synaptic pMad levels (Fig 6C). We found that RNAi-mediated reduction of Mav in the glia induced a significant decrease of synaptic pMad at control NMJs as well as in a *gbb* mutant background. A similar reduction was observed by knockdown of Put, the Mav receptor, in the striated muscle, suggesting that Mav signaling to the muscle may explain its effect on synaptic pMad (see below).

**Fig 7:**
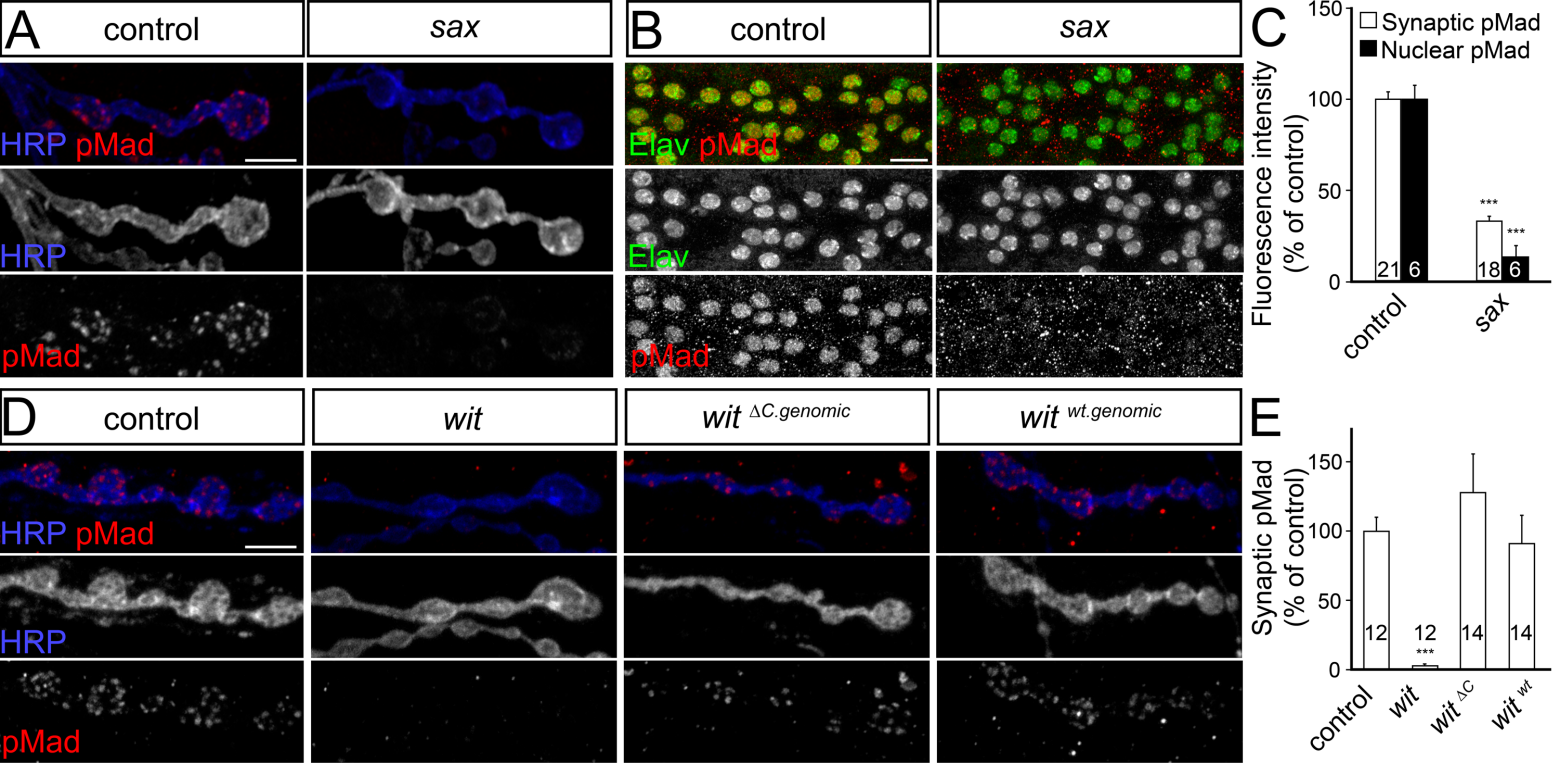
BMP receptors required for synaptic pMad. (A-E) Confocal images of NMJ4 boutons (A, D) and ventral ganglia (B) from larvae of indicated genotypes labeled for pMad (red), Elav (green) and HRP (blue). (A-C) Synaptic and nuclear pMad require the type I BMP receptor Sax (quantified in C). (D-E) Synaptic pMad does not require the LIMK1 binding domain of Wit (quantified in E). Genotypes: control (*w*^*1118*^), *sax* (*sax*^*4/Df*^), *wit* (*wit*^*A12/Df*^), *gbb* (*gbb*^*1/2*^), *gbb; IIA*^*RNAi*^ (*gbb*^*1/2*^; *UAS-GluRIIA*^*RNAi*^/*24B-Gal4*), *gbb; wit* (*gbb*^*1/2*^; *wit*^*A12/Df*^), *wit*^*ΔC*^ (*wit*^*ΔC*.*genomic*^*; wit*^*A12/Df*^), *wit*^*wt*^ (*wit*^*genomic*^*; wit*^*A12/Df*^). Error bars indicate SEM. ***; p<0.001. Scale bars: 5 μm (A, D), 10 μm (B).

In *Drosophila*, two type I receptors, Tkv and Sax, transduce the BMP-type signals. At the NMJ, both Tkv and Sax are required for nuclear pMad accumulation and NMJ growth [17]. Furthermore, excess activated Tkv in the motor neurons induces increased synaptic pMad [45], and excess activated Tkv and Sax restores the pMad signals at *impß11* NMJs [42]. We found that Sax is also required for synaptic pMad accumulation (Fig 7A). *sax* null third instar mutants (*sax*^*4/Df*^) showed dramatically reduced levels of synaptic pMad and practically no detectable nuclear pMad signals above background. Thus, similar to Wit, Sax appears to be required for both nuclear and synaptic pMad accumulation.

In flies, as well as vertebrates, Wit has a large intracellular domain that binds and signals through LIM kinase 1 (LIMK1) to regulate synapse stability and activity-dependent synaptic growth [20,21]. To test whether LIMK1 influences the local pMad accumulation we examined the NMJs of *wit*^*ΔC genomic*^ animals, which lack the LIMK1-binding intracellular part of Wit [15]. Synaptic pMad was restored at *wit* mutant NMJs by either control or *wit*^*ΔC*^ genomic transgenes (Fig 7D and 7E). Thus, Wit is essential for both nuclear and synaptic pMad accumulation, but neither of these functions requires its interaction with LIMK1 [14,15]. In contrast, Gbb is required for nuclear pMad and the transcriptional control of BMP target genes, but appears to be dispensable for pMad accumulation at active zones. This implies that the differences between the *wit* and *gbb* mutant NMJs should reflect, at least in part, a role for synaptic pMad during NMJ development.

### Synaptic pMad and the distribution of iGluR subtypes

**Fig 8:**
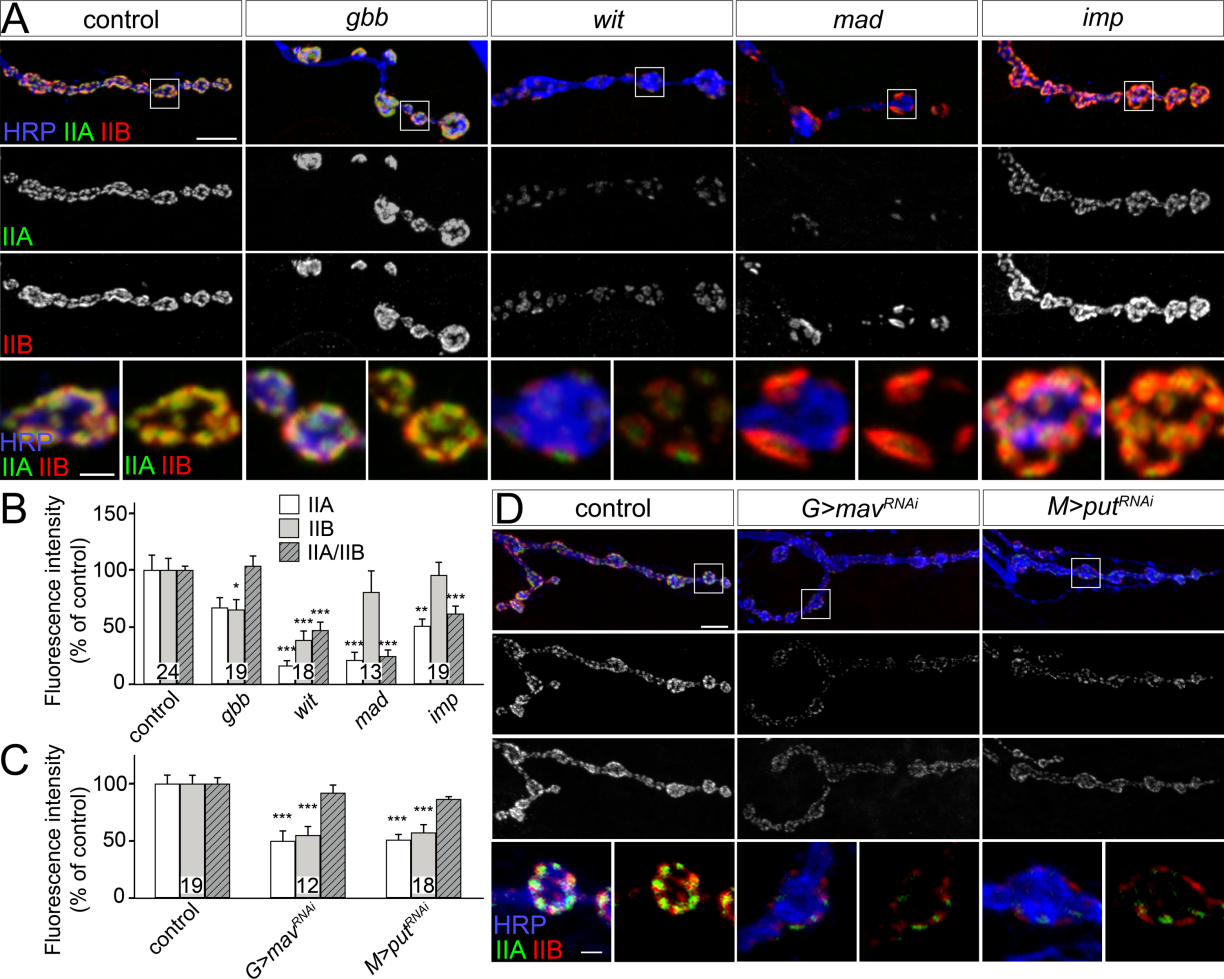
BMP signaling influences iGluR subtypes distribution. (A-D) Confocal images of NMJ4 boutons from third instar larvae of indicated genotypes labeled for GluRIIA (green), GluRIIB (red), and HRP (blue) (quantified in B-C). The relative intensity of postsynaptic GluRIIA and GluRIIB signals decreases unequally in mutants lacking synaptic pMad, and induces a reduction in IIA/IIB ratio, except for *gbb* mutants (A-B). Equal reduction of GluRIIA and GluRIIB signals (and thus normal IIA/IIB ratio) is found in larvae with Mav-depleted glia and Put-depleted muscle (C-D). Genotypes: control (*w*^*1118*^), *gbb* (*gbb*^*1/Df*^), *wit* (*wit*^*A12/Df*^), *mad* (*mad*^*12/Df*^), *imp* (*imp*^*24/70*^), *G>mav*^*RNAi*^ *(repo-Gal4/UAS-mav*^*RNAi*^*)*, *M>put*^*RNAi*^ *(G14-Gal4/UAS-put*^*RNAi*^*)*. Error bars indicate SEM. ***; p<0.001, **; p<0.01, *; p<0.05. Scale bars: 5 μm and 1 μm (details).

Mutations in either *wit* or *gbb* induce severe deficits in NMJ growth and function, but only *wit* mutant NMJs have reduced mini amplitude, or quantal size, the postsynaptic response to the spontaneous fusion of a single synaptic vesicle [14,19]. In contrast, *gbb* mutants have relatively normal quantal size [16,53]. Previous studies established that a key determinant of quantal size is the dose of synaptic GluRIIA versus GluRIIB (IIA/IIB) [25,26]. Therefore, the difference in quantal size in *wit* and *gbb* mutants may arise from different IIA/IIB synaptic composition. We tested this prediction by examining the relative GluRIIA and GluRIIB signal intensities at mutant NMJs. We found that the GluRIIA and GluRIIB synaptic signals were reduced by more than 30% (to 67 ± 8% and 65 ± 9% respectively of controls, n = 19) at *gbb* mutant NMJs, but the IIA/IIB ratio remained normal (Fig 8A and 8B). In contrast, the GluRIIA and GluRIIB signals were severely and unequally reduced in *wit* mutants, such as that the IIA/IIB ratio was reduced to 47 ± 7%, n = 18. A similar, asymmetrical reduction of GluRIIA and GluRIIB synaptic signals and a decreased IIA/IIB ratio was also apparent at *mad* mutant NMJs (Fig 8A and [54]). This is consistent with a small reduction in quantal size observed in *mad* hypomorphs or dominant negative allelic combinations [17,24]. Interestingly, a subtle reduction in the IIA/IIB ratio was also observed at *imp* mutant NMJs, consistent with the previously described 20% decrease in quantal size [42]. Overall, the differences in GluRIIA levels were smaller than those observed for synaptic pMad (Figs 3 and 7 and [12,42]). This may be due to GluRIIA receptors present at synaptic sites but in configurations that cannot trigger local pMad accumulation [12,55].

In contrast, depletion of Mav in the glia produced reduction of both GluRIIA and GluRIIB subunits but did not significantly alter the postsynaptic IIA/IIB ratio (Fig 8C and 8D). The same result was found when Put was depleted in the striated muscle. This severe reduction in the synaptic distribution of both GluRIIA and GluRIIB subunits is reminiscent of Activin-type signaling [54]. It has been shown that motor neuron-derived Activin signals via Baboon, the type I TGF-β receptor, and Smox/dSmad2, the pathway effector, to regulate the expression of GluRIIA and GluRIIB in the muscle. In the absence of Activin pathway components, GluRIIA and GluRIIB transcripts are dramatically reduced, and GluRIIA synaptic distribution is further diminished via post-translational mechanisms. The similarities between Mav/Put and Act/Babo/Smox signaling pathways suggest that these pathways may share common TGF-β receptors (i.e. Put) and converge, at least in part, onto subsets of transcriptional targets, such as GluRIIA and GluRIIB. While these data do not exclude a more direct role for Mav (or Act) in the synaptic accumulation of pMad, these pathways likely influence local pMad indirectly, via postsynaptic GluRIIA, which is both required and sufficient for synaptic pMad accumulation (Fig 3).

**Fig 9:**
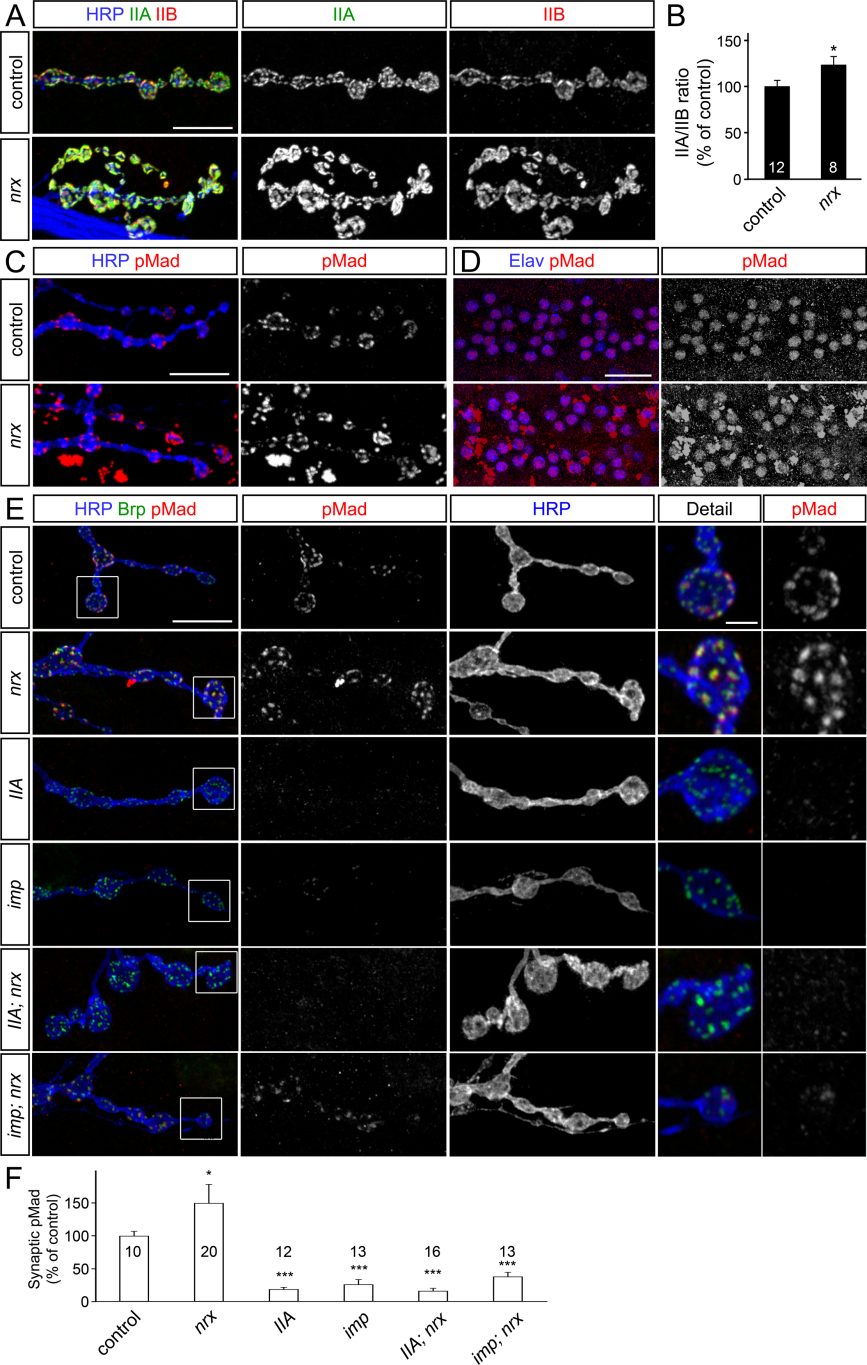
Neurexin limits the synaptic accumulation of pMad. (A) Confocal images of NMJ4 boutons from third instar larvae labeled for GluRIIA (green), GluRIIB (red), and HRP (blue) (quantified in (B)). The Nrx-depleted NMJs have larger boutons, with increased GluRIIA and GluRIIB signals, and 25% increase in the IIA/IIB ratio. (C-D) pMad levels are increased at *nrx* mutant NMJs (C), but not in the motor neuron nuclei (D). (E-F) Confocal images of NMJ4 boutons from larvae of indicated genotypes labeled for pMad (red), HRP (blue) and Brp (green). The increased accumulation of pMad at *nrx* mutant NMJs is suppressed by loss of GluRIIA or Imp. Some synaptic pMad is observed in *imp; nrx* double mutants, but levels remain greatly reduced compared to control, matching the levels seen in *imp* mutants (quantified in F). Genotypes: control (*w*^*1118*^), *nrx* (*nrx*^*273/Df*^), *IIA; nrx* (*GluRIIA*^*SP16/Df*^*; nrx*^*273/Df*^), *imp* (*imp*^*24/70*^), *imp; nrx* (*imp*^*24/70*^*; nrx*^*273/Df*^). Error bars indicate SEM. ***; p<0.001, *; p<0.05. Scale bars: 10 μm (A, C and E), 20 μm (D) and 1 μm (details).

At the *Drosophila* NMJ, the type-A receptors are the first to arrive at nascent synapses, followed by the type-B receptors, which mark more mature synapses [56]. This ordered incorporation of iGluR subtypes is modulated in part by Neurexin (Nrx) and Neuroligin1 (Nlg1), a pair of conserved adhesion molecules that form trans-synaptic complexes which stabilize synaptic contacts and organize receptor fields [57]. Lack of Nrx or Nlg1 perturbs the dynamics of iGluRs recruitment and stabilization at the fly NMJ and causes morphological and physiological defects [58–60]. We found that the IIA/IIB ratio was increased by 25% at *nrx* mutant NMJs (Fig 9A and 9B). This change is consistent with the increased quantal size reported for the *nrx* mutants [60]. Importantly, synaptic but not nuclear pMad levels were increased by 50% in third instar *nrx* larvae (Fig 9C and 9D). A similar, but less dramatic increase of synaptic pMad was found in *nlg1* mutants. In these mutants pMad immunolabeling produced high background, with patches of pMad-positive areas outside of the motor neuron nuclei and of the synaptic terminals; such patches were not included in our quantifications.

As expected, *IIA; nrx* double mutants showed a complete loss of synaptic pMad without any change in the nuclear pMad levels (Fig 9E). The morphology of *IIA; nrx* double mutant NMJs resembled the *nrx* mutants, with fewer and larger boutons, grouped closer together [60]. Thus, the iGluR subtypes and local pMad do not influence the NMJ morphology of *nrx* mutants. This result is similar to that observed for *nwk* or *hiw* mutants (Fig 3). Unlike *nwk*, which limits Tkv endocytosis and restores local pMad at *imp; nwk* double mutant NMJs, loss of Nrx could not rescue the synaptic pMad in *imp; nrx* mutants (Fig 9E and 9F). This suggests that Nrx influences the local pMad indirectly, perhaps by limiting postsynaptic GluRIIA.

Together our data indicate that postsynaptic GluRIIA and presynaptic BMPRs are key determinants for the accumulation of pMad at active zones. Furthermore, BMP signaling modulators (i.e. Nwk and Imp), acting in the presynaptic compartment, control the levels of synaptic pMad. Intriguingly, increased synaptic pMad (such as in *nrx* mutants) correlates with increased IIA/IIB ratio and increased quantal size, while loss of synaptic pMad (in *imp*, *wit* and *mad* mutants) correlates with a decreased IIA/IIB ratio and reduced quantal size. In contrast, the presence of synaptic pMad even in a transcriptionally impaired BMP mutant (i.e. *gbb*) ensured relatively normal IIA/IIB ratio and quantal size. This tight correlation suggests a feedback mechanism whereby active postsynaptic GluRIIA receptors induce the accumulation of pMad at active zones, which in turn promotes the stabilization of GluRIIA receptors at postsynaptic sites. In this scenario, selective disruption of synaptic pMad should “destabilize” the GluRIIA receptors and cause decreased IIA/IIB ratio.

### A positive feedback connects postsynaptic GluRIIA and local pMad

To test for such a positive feedback loop we have to disrupt the local pMad accumulation without affecting the canonical BMP signaling and transcription of BMP target genes. This precludes the use of any BMP signaling components or known BMP modulators, as any such manipulations will affect both local and transcriptional functions of BMP pathway. Previous studies demonstrate that Mad phosphorylation at S25 by Nemo kinase, a MAPK-related kinase, promotes nuclear export of pMad in heterologous cells [61], whereas *nemo* mutant NMJs have increased local pMad [62]. Nemo does not appear to interfere with the ability of BMP/BMPR complexes to phosphorylate Mad at its C-terminal residues; instead, Nemo influences the subcellular distribution of Mad irrespective of its BMP-dependent phosphorylation status. Interestingly, *nemo* mutants have reduced synaptic pMad and neuronal overexpression of activated Tkv, but not Mad, could rescue this deficit, suggesting that BMPRs become limiting in *nemo* mutants [62]. Since the levels of BMPR are tightly controlled [45], and lack of S25 phosphorylation (in *nemo* mutants) increases the synaptic pMad and promotes the pMad-BMPRs association, Nemo-dependent phosphorylation may provide a means for regulating the stability of pMad-BMP/BMPR complexes. This predicts that S25 and (Nemo-phosphorylated) pS25 will have opposing effects on the formation and stabilization of Mad-BMP/BMPR complexes.

**Fig 10:**
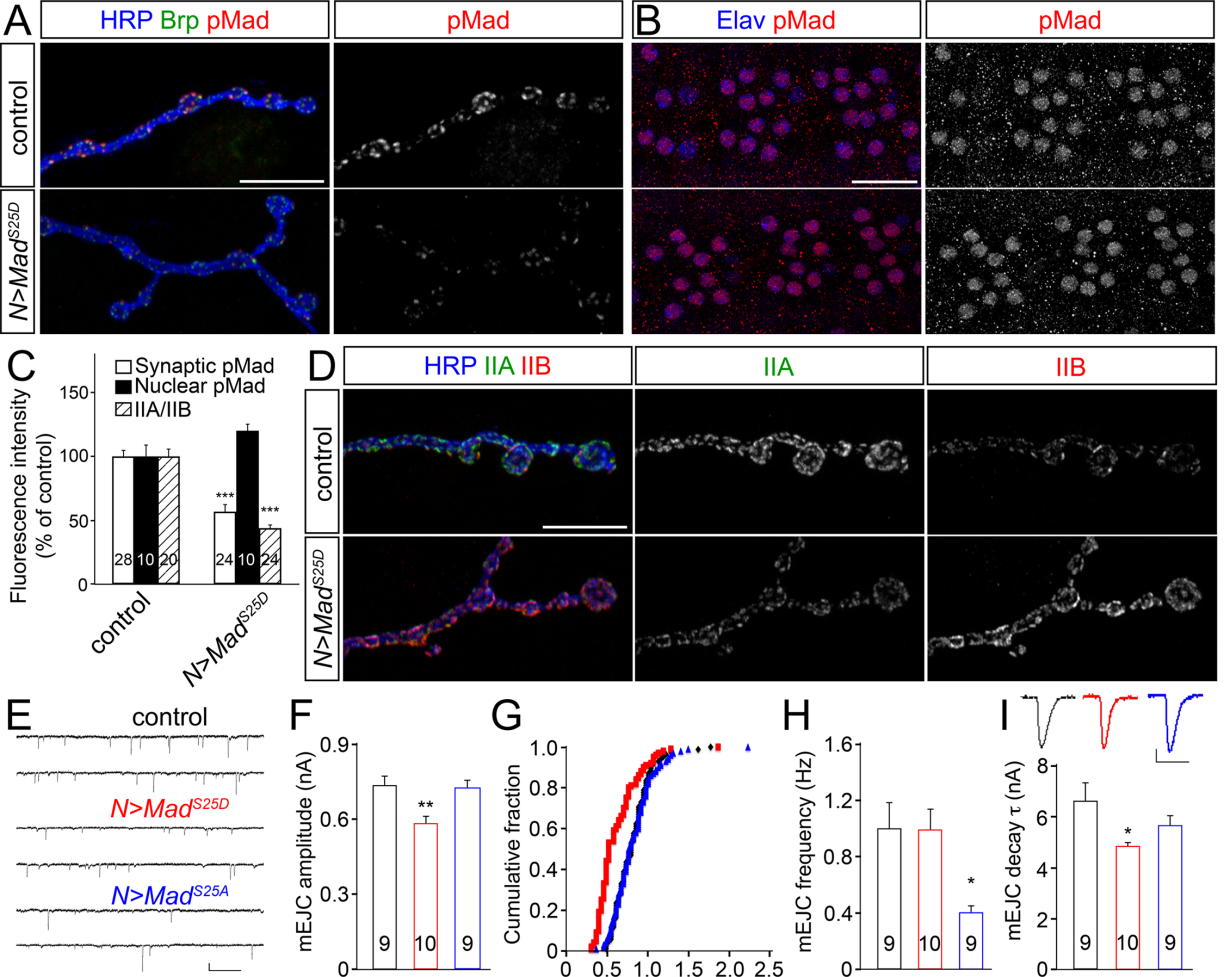
Excess phosphomimetic Mad reduces postsynaptic GluRIIA. (A-D) Confocal images of NMJ4 boutons (A, D) and ventral ganglia (B) (quantified in C) from control and third instar larvae with a phosphomimetic Mad variant overexpressed in motor neurons (*N>Mad*^*S25D*^). Neuronal expression of Mad^S25D^ greatly reduces the accumulation of synaptic pMad (A), but does not affect the nuclear pMad levels (B). Excess presynaptic Mad^S25D^ induces a reduction of GluRIIA synaptic signals (green) and an increase of GluRIIB (red) relative to HRP (blue) (D). Scale bars: 10 μm (A and D) and 20 μm (B). (E-I) TEVC recordings from muscle 6, segment A3, of control and third instar larvae with excess presynaptic Mad^S25D^ (*N>Mad*^*S25D*^) or Mad^S25A^ (*N>Mad*^*S25A*^). (E) Representative traces of spontaneous junction currents recorded at 0.5 mM Ca^2+^. Summary graphs showing the mean amplitude (F) cumulative probability (G) mean frequency (H) and decay time constant (I) of mEJCs. The mEJC amplitude and decay constant were reduced when Mad^S25D^ was overexpressed in the motor neurons. Overexpressing Mad^S25A^ did not affect mEJC amplitude or decay constant but showed a reduction in mEJC frequency. Scale bars: 0.5 nA/500 ms (E) and 0.2 nA/25 ms (I). Genotypes: control (*380-Gal4/Y*), *N>Mad*^*S25D*^ (*380-Gal4/Y; +; UAS-Mad*^*S25D*^*/+*), *N>Mad*^*S25A*^ (*380-Gal4/Y; +; UAS-Mad*^*S25A*^*/+*). Error bars indicate SEM. ***; p<0.001, **; p<0.01, *; p<0.05.

We reasoned that overexpression of a Nemo-phosphomimetic Mad variant (S25D) in the motor neurons should not affect the nuclear pMad pool since excess Mad^S25D^ will be efficiently exported from the motor neuron nuclei. However, at active zones, excess Mad^S25D^ will compete with the endogenous Mad for BMPR-mediated phosphorylation, but will presumably dissociate from the presynaptic BMP/BMPR complexes, thus diminishing the local pMad accumulation. Indeed, we found that neuronal overexpression of a *Mad*^*S25D*^ transgene induced a minimal increase in the nuclear pMad pool (20% more than controls, n = 10) but significantly reduced the synaptic pMad levels (to 55% of control, n = 24) (Fig 10A, 10B and 10C). By comparison, neuronal overexpression of wild-type Mad, or the phospho-mutant variant Mad^S25A^, induced a 4-fold increase in nuclear pMad and no change in the synaptic pMad levels (Figs 4 and S2). More importantly, neuronal expression of Mad^S25D^ caused a drastic reduction in the postsynaptic GluRIIA levels (Fig 8C and 8D). At the same time the GluRIIB levels were increased, likely because of the competition between GluRIIA and GluRIIB for the essential iGluR subunits [27]. The excess neuronal Mad^S25D^ did not influence the NMJ morphology and growth and did not affect the Twit-GFP levels, indicating normal transcriptional regulation in response to the canonical BMP signaling. The number of synaptic contacts and the iGluR receptor fields, as visualized by juxtaposed Brp and GluRIIC signals, also appeared normal (S3 Fig). The only change we could detect in animals with excess presynaptic Mad^S25D^/reduced synaptic pMad was a reduced IIA/IIB ratio.

To examine whether this change in the distribution of iGluR subtypes influences NMJ function, we performed electrophysiology recordings of spontaneous junction currents and potentials from muscle 6 of control and third instar larvae expressing various *Mad* transgenes in motor neurons (Figs 10E–10I and S4). Consistent with the reduced IIA/IIB ratio observed, neuronal overexpression of Mad^S25D^, but not Mad^S25A^, caused a 22% reduction in mEJC amplitude (0.58 ± 0.03 nA for Mad^S25D^ vs control 0.74 ± 0.04 nA, or Mad^S25A^ 0.73 ± 0.03 nA, *p < 0*.*01*). Furthermore, the decay time constant was decreased when Mad^S25D^ was overexpressed in the motor neurons (to 4.85 ± 0.14 ms for Mad^S25D^, comparing to control 6.61 ± 0.74 ms, and Mad^S25A^ 5.66 ± 0.40 ms, respectively; *p < 0*.*05*). Since GluRIIB-containing receptors desensitize much faster that the GluRIIA [26], these data are consistent with the observed shift towards more GluRIIB postsynaptic receptors at excess Mad^S25D^. Overexpression of the phospho-mutant variant Mad^S25A^ had no significant effect on the mEJC amplitude and decay constant, but produced a strong reduction of mEJC frequency. This may be due to excess nuclear Mad^S25A^ and perturbed expression of BMP target genes, including *twit*, which encodes a modulator of mini frequency.

Similarly, neuronal overexpression of Mad^S25D^, but not Mad^S25A^, caused a 25% reduction in mEJP amplitude (*p = 0.020, S4 Fig). We found no change in the resting potential and input resistance in these larvae. As in the case of *GluRIIA* mutants, the amplitude of evoked junctional potentials remained normal at NMJs with excess neuronal Mad^S25D^ (p = 0.555, S4 Fig), demonstrating a compensatory increase in quantal content, the number of vesicles released in response to each action potential. We estimated the quantal content by dividing the mean EJP amplitude to mean mEJP and found an 80% increase in quantal content (*p = 0.032, S4 Fig) at NMJs with excess Mad^S25D^. This indicates a robust presynaptic response to the Mad^S25D^-induced reduction of IIA/IIB ratio.

These findings suggest that diminished synaptic pMad in the motor neurons causes a reduction of postsynaptic GluRIIA and induces a change in the synaptic accumulation of iGluR subtypes towards more type-B receptors. The reduction of IIA/IIB ratio, evident in histology as well as electrophysiology experiments, induced a significant increase in quantal content compared with control. Such compensatory response in presynaptic transmitter release is characteristic of low levels of postsynaptic GluRIIA [26]. Thus, synaptic pMad accumulates in response to active GluRIIA and, in turn, appears to stabilize the type-A receptors at synaptic sites. This positive feedback could shape the synaptic composition for iGluRs as a function of type-A receptor activity.

## Discussion

BMPs fulfill multiple functions during NMJ development via canonical and noncanonical pathways. In motor neurons, signaling by Gbb triggers a canonical BMP signaling that regulates transcription of BMP target genes and a noncanonical BMP pathway that connects Wit with LIMK1 and the cytoskeleton. Here we describe a novel non-canonical BMP pathway, which induces selective accumulation of pMad at presynaptic sites. This pathway does not require Gbb, but depends on BMP receptors Wit and Sax and postsynaptic GluRIIA. This novel pathway does not contribute to the NMJ growth and instead appears to set up a positive feedback loop that modulates the postsynaptic distribution of type-A and type-B receptors as a function of synapse activity.

### Multiple BMP signaling pathways at the *Drosophila* NMJ

At the *Drosophila* NMJ, BMP signaling controls NMJ growth and promotes synapse homeostasis [14–16,63]. BMP fulfills all these functions via canonical and noncanonical pathways. Canonical BMP signaling activates presynaptic transcriptional programs with distinct roles in the structural and functional development of the NMJ [18,19]. For example, the BMP pathway effector Trio can rescue NMJ growth in BMP pathway mutants, but does not influence synapse physiology, whereas Twit can partially restore the mini frequency but has no effect on NMJ growth. It has been shown that both muscle and neuron derived Gbb are required for the structural and functional integrity of NMJ, and multiple mechanisms that regulate Gbb expression, secretion and extracellular availability have been described [41,53,64,65]. Binding of Gbb to its receptors also triggers a noncanonical, Mad-independent pathway that requires the C-terminal domain of Wit. This domain is conserved among *Drosophila* Wit and vertebrate BMPRII and functions to recruit and activate cytoskeletal regulators such as LIMK1 [66,67]. In flies, Wit-mediated activation of LIMK1 mediates synapse stability and enables rapid, activity-dependent synaptic growth [20,21].

In this study we uncovered a novel, noncanonical BMP pathway that triggers accumulation of presynaptic pMad in response to postsynaptic GluRIIA receptors. This pathway requires Wit and Sax, suggesting that various BMP pathways compete for shared components. Super resolution imaging mapped the pMad domains at active zones, in close proximity to the presynaptic membrane. These domains concentrate the pMad immunoreactivities into thin discs that reside mostly within individual synapse boundaries. The size and shape of pMad domains suggest that pMad could associate with membrane-anchored complexes at the active zone. Since BMP signals are generally short lived [33,34,68], these pMad domains likely represent pMad that, upon phosphorylation, remains associated with the BMP/BMPR kinase complexes at synaptic sites. Alternatively, pMad may accumulate in synaptic aggregates that protect it from dephosphorylation. While we cannot exclude the second possibility, several lines of evidence support the first scenario. First, excess Mad cannot increase the levels of synaptic pMad (Fig 4). Second, neuronal expression of activated Tkv/Sax but not Mad can restore the synaptic pMad at *impß11* mutant NMJs [42]. Finally, during neural tube closure, junctional pSmad1/5/8 and its association with PAR complexes depend on BMPs [8]. Previous studies indicate a reduction of synaptic pMad signals in response to muscle-specific Mad RNAi [9,41]. We too have observed such a reduction (S5 Fig). In addition, we found a significant decrease of postsynaptic IIA/IIB ratio in Mad-depleted muscles: GluRIIA and GluRIIB synaptic levels were reduced to 49% and respectively 78% of control (n = 21). Since GluRIIA is key to the synaptic pMad accumulation we suspect that the muscle Mad RNAi phenotype is due to perturbation in synaptic GluRIIA levels, perhaps by interference with the Activin signaling pathway (see below).

How are the BMP/BMPR complexes stabilized at synaptic sites? Studies on single receptors demonstrate that the confined mobility of BMPRI on the plasma membrane is key to stabilize BMP/BMPR complexes and differentially stimulate canonical versus noncanonical signaling [69]. Direct interactions between phosphorylated Smad5 and the Par3-Par6-aPKC polarity complex occur at the apical junctions [8]. Similarly, synaptic pMad, which remains associated with BMP/BMPR complexes, may engage in interactions that restrict the mobility of BMP/BMPR complexes on the presynaptic membrane. Nemo-mediated phosphorylation of Mad-S25 could disrupt the pMad/BMPR association and expose the BMP/BMPR complexes, so they could dissociate and/or be internalized. The heteromeric BMPR complexes are transient; ligand binding greatly increases their lifespan and stability [70]. Albeit Gbb is not essential for synaptic pMad, it may act redundantly with other ligands to stabilize BMP/BMPR local complexes. Several ligands secreted in the synaptic cleft have been shown to bind and signal via BMPRII; they include glia secreted Maverik [41,71], Myoglianin, which could be secreted from muscle and/or glia [72,73], and Activins [74]. However, these ligands also appear to signal via a canonical Activin pathway, which regulates the postsynaptic GluRIIA/GluRIIB abundance at the *Drosophila* NMJ [54]. Alterations in the Activin signaling pathway drastically alter the synaptic recruitment of both iGluR subtypes, in particular the GluRIIA, which controls synaptic pMad, making it difficult to identify the nature and the directionality of the signaling molecule(s) involved in the synaptic pMad accumulation. Interestingly, all of these ligands are substrates for BMP-1/Tolloid-type enzymes, which control their activity and distribution [75]. Treatments that induce long-term stimulation up-regulate a BMP-1/Tolloid homolog in *Aplysia* neurons [76].

An intriguing aspect of this novel BMP pathway is the dependence on active postsynaptic GluRIIA, which is both required and sufficient for pMad accumulation at active zones. Since pMad and BMP/BMPR complexes cluster at synaptic sites (Fig 1 and [45]), we speculate that trans-synaptic complexes may couple postsynaptic type-A glutamate receptors with presynaptic BMP/BMPRs. The synaptic cleft is 200 Å; the iGluR tetramer expands 135 Å in the synaptic cleft [77], and the BMP/BMPR complexes ~55 Å [78,79]. The iGluRs auxiliary subunit Neto has extracellular CUB and LDLa domains predicted to expand 120–130 Å in the synaptic cleft, based on related structures. CUB domains are BMP binding motifs [80] that may localize BMP activities and/or facilitate ligand binding to BMPRs. In this model, Neto provides the link between postsynaptic GluRIIA and presynaptic BMP/BMPR complexes. During receptors gating cycle, the iGluRs undergo corkscrew motions that shorten the channels as revealed by cryo-electron microscopy [81]. Such movements may influence the stability of trans-synaptic complexes and allow synaptic pMad to function as a sensor for GluRIIA activity [12].

### A positive feedback loop sculpts postsynaptic composition

While more components of this novel pathway remain to be determined, it is clear that this pathway does not contribute to NMJ growth and instead has a critical role in synapse maturation. Unlike canonical BMP signaling, loss of local pMad induces minor reductions in bouton number [12,25,82] and does not rescue the NMJ overgrowth of endocytosis mutants (Fig 3). Local pMad accumulates independently of Wit-mediated LIMK1 activation and does not appear to influence synapse stabilization; in fact, *nrx* mutants have synapse adhesion defects [60] but show increased synaptic pMad levels (Figs 7 and 9). The striking correlation between synaptic pMad levels and GluRIIA activity, together with previous findings that GluRIIA activity and gating behavior directly impacts receptor mobility and synaptic stabilization [26,31] suggest a positive feedback mechanism in which active GluRIIA receptors induce stabilization of BMP/BMPR complexes at synaptic sites which, in turn, promote stabilization of type-A receptors at PSDs. In this scenario, presynaptic pMad marks active BMP/BMPR complexes and acts to maintain the local BMP/BMPR complexes in large clusters that evade endocytosis. Selective disruption of local pMad via a neuronal dominant-negative Mad^S25D^ presumably destabilizes the large presynaptic BMP/BMPR clusters and causes a significant reduction in the IIA/IIB ratio and quantal size (Fig 10).

This positive feedback couples synaptic activity with synapse development and is controlled by (1) active GluRIIA receptors, (2) presynaptic BMP receptors, Wit, Sax, and likely Tkv, (3) mechanisms regulating BMPR heteromers assembly, endocytosis and turnover, and (4) the ability of pMad to remain associated with its own kinase upon phosphorylation. Perturbations of any of these components trigger variations in local pMad levels accompanied by changes in the IIA/IIB ratio and/or quantal size. For example, *nemo* mutants have increased synaptic pMad levels and increased mEJCs [62], while *imp* mutants have decreased synaptic pMad levels and decreased mEJPs (Fig 3 and [42]). The assembly and function of these putative trans-synaptic complexes, in particular ligand availability, should be influenced by the composition and organization of the synaptic cleft. Indeed, local pMad and quantal size are increased in mutants lacking heparan sulfate 6-O-endosulfatase (*sulf1)*, or 6-O-sulfotransferase (*hs6st*) [65]. Since this Mad-dependent, noncanonical pathway shares components with the other BMP signaling pathways, the balance among different BMP pathways may coordinate the NMJ development and function.

The complexity of BMP signaling at the *Drosophila* NMJ is reminiscent of the neurotrophin-regulated signaling in vertebrate systems (reviewed in [83]). Neurotrophins were first identified as neuronal survival factors. Like BMPs, they are secreted as pro-proteins that must be processed to form mature ligands. The active dimers bind to transmembrane kinase receptors and induce their activation through trans-phosphorylation. Neurotrophin/receptor complexes are internalized and transported along axons to the cell soma [84]; signaling in the cell soma controls gene expression and promotes neuronal differentiation and growth. In addition, local neurotrophin signaling regulates growth cone motility, enhances the presynaptic release of neurotransmitter and mediates activity-dependent synapse formation and maturation (reviewed in [85]). At the *Drosophila* NMJ, several neurotrophins have been implicated in neuron survival, axon guidance and synapse growth [86–88]. It will be interesting to test for the crosstalk between neurotrophin and BMP signaling at these synapses.

To our knowledge, the novel noncanonical BMP pathway reported here is the first example of a BMP pathway triggered by selective neurotransmitter receptors and influencing receptor distribution at PSDs. We expect that some of these functions will apply to mammalian glutamatergic synapses: First, as indicated in the Allen Brain Atlas, glutamate receptors and Neto proteins are widely expressed in mammalian brain structures where BMPs, BMPRs and Smads are expressed. Second, BMPs have been shown to rapidly potentiate glutamate-mediated currents in human retina neurons, presumably via a noncanonical pathway [89]. Finally, mice lacking Chordin, a BMP antagonist, have enhanced paired-pulse facilitation and LTP and show improved learning in a water maze test [90]. Such changes could not be explained by Smad-dependent transcriptional responses and were not accompanied by structural alterations in synapse morphology. Instead, presynaptic noncanonical BMP pathway may influence the activity of postsynaptic glutamate receptors by modulating their synaptic distribution and stability.

## Materials and Methods

### Fly stocks

*Drosophila* stocks used in this study are as follows: *GluRIIA*^*SP16*^, *Df(2L)cl*^*h4*^, and *UAS-GluRIIA* [25] (from A. DiAntonio, Washington University); *hiw*^*ND8*^ [46]; *impß11*^*24*^ and *impß11*^*70*^ [42]; *twit*^*MI06552*^ [51]; *mad*^*12*^ [91]; *mad* deficiency *Df(2L)C28* [92]; *UAS-Mad-GFP* [9] (from M. Gonzalez-Gaitan, University of Geneva); *UAS-Mad-Myc [62]*; *UAS-T7-Mad*^*S25A*^ [61]; *gbb*^*1*^ and *gbb*^*2*^ [93]; *sax*^*4*^ [94]; *wit*^*ΔC*.*genomic*^, *wit*^*A12*^ and *wit*^*B11*^ [15] (from M. O’Connor, University of Minnesota); *nwk*^*1*^, *nwk*^*γ3*^ [95] (from K. O’Connor-Giles, University of Wisconsin); *nrx*^*273*^ and *nlg1*^*Δ46*^ [96] (from B. Mozer, NIH); *BG380-Gal4* [97]; *elav-Gal4* (BL-8760); *24B-Gal4* (BL-1716); *G14-Gal4* and *MHC-Gal4* (from C. Goodman, University of California at Berkeley). For *UAS-T7-Mad*^*S25D*^, the T7-tagged Mad^S25D^ [61] was cloned into pUAST and transgenic lines were generated by germline transformation (BestGene). For *RNAi*-mediated knockout we used UAS-put-RNAi and UAS-mad-RNAi (ID 848 and respectively 12635, Vienna Drosophila RNAi Center) and TRiP lines generated by the Transgenic RNAi Project, Harvard Medical School, *GluRIIA* (*P[TRiP*.*JF02647]attP2*), and *mav* (*P[TRiP*. *HMS01125]attP2* and *P[TRiP*. *GL01025]attP40)*.

The flies were reared on Jazz-Mix food (Fisher Scientific). To control for larvae crowding, 8–10 females were crossed with 5–7 males per vial and were passed to fresh vials every 3 days. For rearing on yeast, embryos were collected on grape agar plates for 24 hours, incubated at 25°C for 24 hours and then 50 first instar larvae of appropriate genotype were transferred to a vial of standard fly food containing a 20 mm^2^ paper saturated with 20% (w/v) active dry baker’s yeast in water. Larvae remained on paper and did not burrow into food. Fresh yeast solution was added daily to keep paper saturated. Larvae were kept on yeast at 25°C until reaching third instar stage.

### Immunohistology

Larvae were dissected as described previously in ice-cooled Ca^2+^-free HL-3 solution [98,99]. The samples were fixed in either 4% formaldehyde (Polysciences, Inc.) for 25 min or in Bouin’s fixative (Bio-Rad) for 3 min and washed in PBS containing 0.5% Triton X-100. Primary antibodies from Developmental Studies Hybridoma Bank were used at the following dilutions: mouse anti-GluRIIA (MH2B), 1:200; rat anti-Elav (7E8A10), 1:200; mouse anti-Bruchpilot (Brp) (Nc82), 1:200. Other primary antibodies were as follows: rabbit anti-phosphorylated Mothers against decapentaplegic (pMad), 1:500, (a gift from Carl Heldin) [100]; rabbit anti-pSmad3, 1:500, (Epitomics, [10]); FITC-, rhodamine-, and Cy5- conjugated goat anti-HRP, 1:1000 (Jackson ImmunoResearch Laboratories, Inc.); rabbit anti-GFP, 1:250 (Abcam); rat anti-Neto, 1:1000 [37]; Cy5- conjugated goat anti-HRP, 1:1000 (Jackson ImmunoResearch Laboratories, Inc.). The rabbit polyclonal anti-GluRIIB and anti-GluRIIC were generated as previously described [26] against synthetic peptides ASSAKKKKKTRRIEK, and QGSGSSSGSNNAGRGEKEARV respectively (Pacific Immunology Corp). Alexa Fluor 488-, Alexa Fluor 568-, and Alexa Fluor 647- conjugated secondary antibodies (Molecular Probes) were used at 1:400.

Larval filets were mounted in ProLong Gold and brains were mounted in SlowFade Gold (Invitrogen). Samples of different genotypes were processed simultaneously and imaged under identical confocal settings using laser scanning confocal microscopes (CarlZeiss LSM780). Boutons were counted using anti-HRP immunoreactivities. All quantifications were performed while blinded to genotype. The numbers of samples analyzed are indicated inside the bars.

All the pMad data quantified here were obtained using the anti-pMad serum from Carl Heldin. For these analyses, the samples were fixed with either 4% formaldehyde or Bouin fixative as described above, washed extensively in PBS containing 0.5% Triton X-100, marked by distinct cuts per genotype and pooled together in the same tube, and, without any blocking agent, incubated overnight at 4°C with anti-pMad 1:500 plus other relevant primary antibodies. The samples were then washed with PBS containing 0.5% Triton X-100, incubated with secondary antibodies (1:200) and no blocking agent for either 2 hours at room temperature or overnight at 4°C. After extensive washes with PBS containing 0.5% Triton X-100, the samples were mounted as above, then imaged and quantified together.

### Fluorescence intensity measurements

For motor neuron nuclei, confocal regions of interest (ROIs) were determined with Imaris software (Bitplane) by using the “spots” feature to automatically identify motor nuclei using Elav immunoreactivity. Spots were verified manually and mean center intensity for all nuclei in a given sample was recorded. This procedure was repeated for all samples of a given genotype and the mean was used for comparison between genotypes. For NMJ signal quantifications, mean signal intensity within the ROI encompassing the synaptic area was normalized to HRP signal. To determine Brp intensity per puncta, individual puncta within the ROI were manually counted and total Brp intensity was divided by the number of puncta. Student’s *t* test was performed using Sigma Plot (Systat) to evaluate statistical significance. All graphs represent mean value of all samples of the given genotype ± SEM.

### Structural illumination microscopy (SIM)

Samples were prepared as described for immunohistology and mounted using #1.5 cover glasses (Cat. 12-541-B, Fisher Scientific). 5 phases and 3 rotations of 3D SIM images were captured using a Zeiss Elyra microscope. The interval for all z stacks was 110nm. Channels were aligned using parameters obtained from calibration measurements with 100 nm TetraSpeck beads. Zeiss SIM images were taken with a 100X 1.46 NA oil objective and a PCO edge sCMOS camera (16 bit images). Laser power and exposure time were optimized to use a large portion of the camera’s dynamic range while minimizing bleaching. As a part of the reconstruction processing using the Zeiss Zen software, Wiener filtering was carefully optimized to maximize resolution and minimize artifacts. The estimated resolution after reconstruction was ~100 nm lateral and ~250 nm axial. Using the Zeiss Zen software, we generated intensity profiles across structures of interest and exported the table containing the fluorescence intensity as a function of distance. To measure distances we calculated the distances between intensity peaks. Surface rendering was performed using Imaris software.

### Electrophysiology

Recordings were performed on muscle 6, segment A3 of third instar larvae as previously reported [29]. Briefly, wandering third instar larvae were dissected in ice-cold, calcium-free physiological HL-3 saline [98], and immersed in HL-3 containing Ca^2+^ before being shifted to the recording chamber. The calcium-free HL-3 saline contains (in mM): 70 NaCl, 5 KCl, 20 MgCl_2_, 10 HCO3, 5 trehalose, 115 sucrose, 5 HEPES, pH adjusted to 7.2 at room temperature. The recording solution was HL-3 with either 0.4 or 0.5 mM CaCl_2_ as described in the text. Intracellular electrodes (borosilicate glass capillaries of 1 mm diameter) were filled with 3 M KCl and resistances ranged from 12 to 25 MΩ. Recordings were done at room temperature from muscle cells with an initial membrane potential between –50 and –70 mV, and input resistances of ≥ 4 MΩ. For mEJCs recording the muscle cells were clamped to –80 mV. To calculate mean amplitudes and frequency of mEJCs or mEJP, 100–150 events from each muscle were measured and averaged using the Mini Analysis program (Synaptosoft). Minis with a slow rise and falling time arising from neighboring electrically coupled muscle cells were excluded from analysis [101,102]. To measure the decay time constant of mEJCs, 20–30 clear representative events from each recording were averaged and fit by a single exponential function. For evoked EJP recordings, the nerve roots were cut near the exiting site of the ventral nerve cord so that the motor nerve could be picked up by a suction electrode. Following motor nerve stimulation with a suction electrode (100 μs, 5 V), evoked EJPs were recorded. Four to six EJPs evoked by low frequency of stimulation (0.1 Hz) were averaged. Quantal content was calculated by dividing the mean EJP by the mean mEJP. Since data were recorded in low calcium saline (0.4 mM Ca^2+^) no correction was made for nonlinear summation. Statistical analysis used KaleidaGraph 4.5 (Synergy Software).

Electrical signals were recorded with an Axoclamp 2B amplifier (Axon Instruments). The signals were filtered at 1 kHz and digitized at 10 kHz by using an analog-digital converter (Digidata 1440A) and pCLAMP software (version 10.0, Axon Instruments). Data are presented as mean ±SEM. One-way ANOVA followed by a Tukey’s post hoc test was used to assess statistically significant differences among genotypes. Differences were considered significant at p < 0.05.

## Supporting Information

S1 Movie Representative SIM z stack for control NMJ12 bouton. 3D-SIM of NMJ12 boutons from third instar larvae labeled for Brp (green), pMad (red), and Neto (blue).
(MOV)

S2 Movie Representative SIM z stack for the control NMJ12 bouton shown in Fig 1D. (MOV)

S3 Movie SIM z series for an individual synapse from control NMJ12 bouton shown in Fig 1H. (MOV)

S4 Movie Representative SIM z stack for the *gbb* mutant NMJ12 bouton. 3D-SIM of NMJ12 bouton from third instar larvae labeled for Brp (green), pMad (red), and Neto (blue).
(MOV)

S5 Movie SIM z series for the individual synapse from *gbb* mutant NMJ12 bouton shown in Fig 5E, labeled for Brp (cyan), pMad (magenta), and Neto (yellow). Scale bars: 100 nm.
(MOV)

S1 Fig Synaptic pMad is decreased in yeast-fed larvae. (A) (A-B) Confocal images of NMJ4 boutons (A) and ventral ganglia (B) from control and *gbb* mutant larvae immunolabeled for pMad (red), and Brp (green) and HRP (blue) (A) or Elav (blue) (B). Rearing larvae on a yeast diet largely eliminated synaptic pMad in both controls and *gbb* mutants. In contrast, nuclear pMad does not change. (C-D) Quantification of mean intensity for synaptic pMad (C) or nuclear pMad (D). Genotypes: control (*w*^*1118*^), *gbb* (*gbb*^*1*^*/gbb*^*Df*^). Error bars indicate SEM. **; p<0.001. Scale bars: 5 μm (A) and 20 μm (B).
(TIF)

S2 Fig Neuronal expression of phosphomutant Mad increases nuclear but not synaptic pMad. (A-B) Confocal images of NMJ4 boutons (A) and ventral ganglia (B) from control and third instar larvae with a phosphomutant (S25A) Mad variant overexpressed in motor neurons (*N>Mad*^*S25A*^). Neuronal expression of Mad^S25A^ does not affect the synaptic pMad (A) while greatly increases the accumulation of nuclear pMad (B) (quantified in (C-D)). Genotypes: control (*w*^*1118*^), *N>Mad*^*S25A*^ (*380-Gal4/Y; +; UAS-Mad*^*S25A*^*/+)*. Error bars indicate SEM. **; p<0.001. Scale bars: 5 μm (A) or 15 μm (B).
(TIF)

S3 Fig Excess presynaptic Mad^S25D^ does not affect the synaptic contacts or net synaptic iGluRs. Confocal images of NMJ4 from third instar larvae labeled for HRP (blue), Brp (green), and GluRIIC (red). Excess presynaptic Mad^S25D^ does not alter the density and integrity of synaptic contacts as measured by juxtaposed Brp and GluRIIC signals. The net GluRIIC synaptic levels appear normal in animals with excess Mad^S25D^ compared to controls. Genotypes: control (*380-Gal4/Y*), *N>Mad*^*S25D*^ (*380-Gal4/Y; +; UAS-Mad*^*S25D*^*/+)*. Error bars represent SEM. Scale bars: 5 μm and 1 μm (details).
(TIF)

S4 Fig Excess phosphomimetic Mad causes electrophysiological defects consistent with reduced GluRIIA. (A-G) Electrophysiological recordings from muscle 6, segment A3, of control and third instar larvae with excess presynaptic Mad^S25D^ (*N>Mad*^*S25D*^) or Mad^S25A^ (*N>Mad*^*S25A*^). Representative traces of mEJPs are shown in (A) and the results are summarized in (G). The number of NMJs examined is indicated in each bar. The mEJPs amplitude (B-C) but not frequency (D) was reduced when Mad^S25D^ was overexpressed in the motor neurons. However, the EJP amplitude was normal due to a significant increased in quantal content (E-F). The muscle resting potential and the input resistance were not affected. Genotypes: control (*380-Gal4/Y*), *N>Mad*^*S25D*^ (*380-Gal4/Y; +; UAS-Mad*^*S25D*^*/+*), *N>Mad*^*S25A*^ (*380-Gal4/Y; +; UAS-Mad*^*S25A*^*/+*). Error bars indicate SEM. *; p<0.01.
(TIF)

S5 Fig Muscle-specific Mad-RNAi causes severely reduced GluRIIA. (A-B) Confocal images of NMJ4 boutons from larvae of indicated genotypes labeled for GluRIIA (green), pMad or GluRIIB (red) and HRP (blue). Mad-depleted muscles have mildly reduced synaptic pMad but severely disrupted GluRIIA synaptic levels (quantified in C). Genotypes: control (*UAS-Mad*^*RNAi*^*/+)*, *M>Mad*^*RNAi*^ (*24B-Gal4/UAS-Mad*^*RNAi*^). Error bars indicate SEM. ***; p<0.001.
(TIF)
