# Correction: A Novel, Noncanonical BMP Pathway Modulates Synapse Maturation at the *Drosophila* Neuromuscular Junction

**DOI:** 10.1371/journal.pgen.1007343

**Published:** 2018-05-11

**Authors:** Mikolaj J. Sulkowski, Tae Hee Han, Carolyn Ott, Qi Wang, Esther M. Verheyen, Jennifer Lippincott-Schwartz, Mihaela Serpe

S1–S5 Movies do not download correctly. The authors have provided corrected S1–S5 Movies, available below. We advise using the media player QuickTime Player 7 for best results.

## Supporting information

S1 Movie Representative SIM z stack for control NMJ12 bouton. 3D-SIM of NMJ12 boutons from third instar larvae labeled for Brp (green), pMad (red), and Neto (blue).
(MOV)

S2 Movie Representative SIM z stack for the control NMJ12 bouton shown in Fig 1D. (MOV)

S3 Movie SIM z series for an individual synapse from control NMJ12 bouton shown in Fig 1H. (MOV)

S4 Movie Representative SIM z stack for the *gbb* mutant NMJ12 bouton. 3D-SIM of NMJ12 bouton from third instar larvae labeled for Brp (green), pMad (red), and Neto (blue).
(MOV)

S5 Movie SIM z series for the individual synapse from *gbb* mutant NMJ12 bouton shown in Fig 5E, labeled for Brp (cyan), pMad (magenta), and Neto (yellow). Scale bars: 100 nm.
(MOV)
