# Supplementary figures and images for: A Novel, Noncanonical BMP Pathway Modulates Synapse Maturation at the Drosophila Neuromuscular Junction
Source: PLoS Genet. 2016 Jan 27;12(1):e1005810. doi: 10.1371/journal.pgen.1005810 (PMC4729469; doi:10.1371/journal.pgen.1005810)

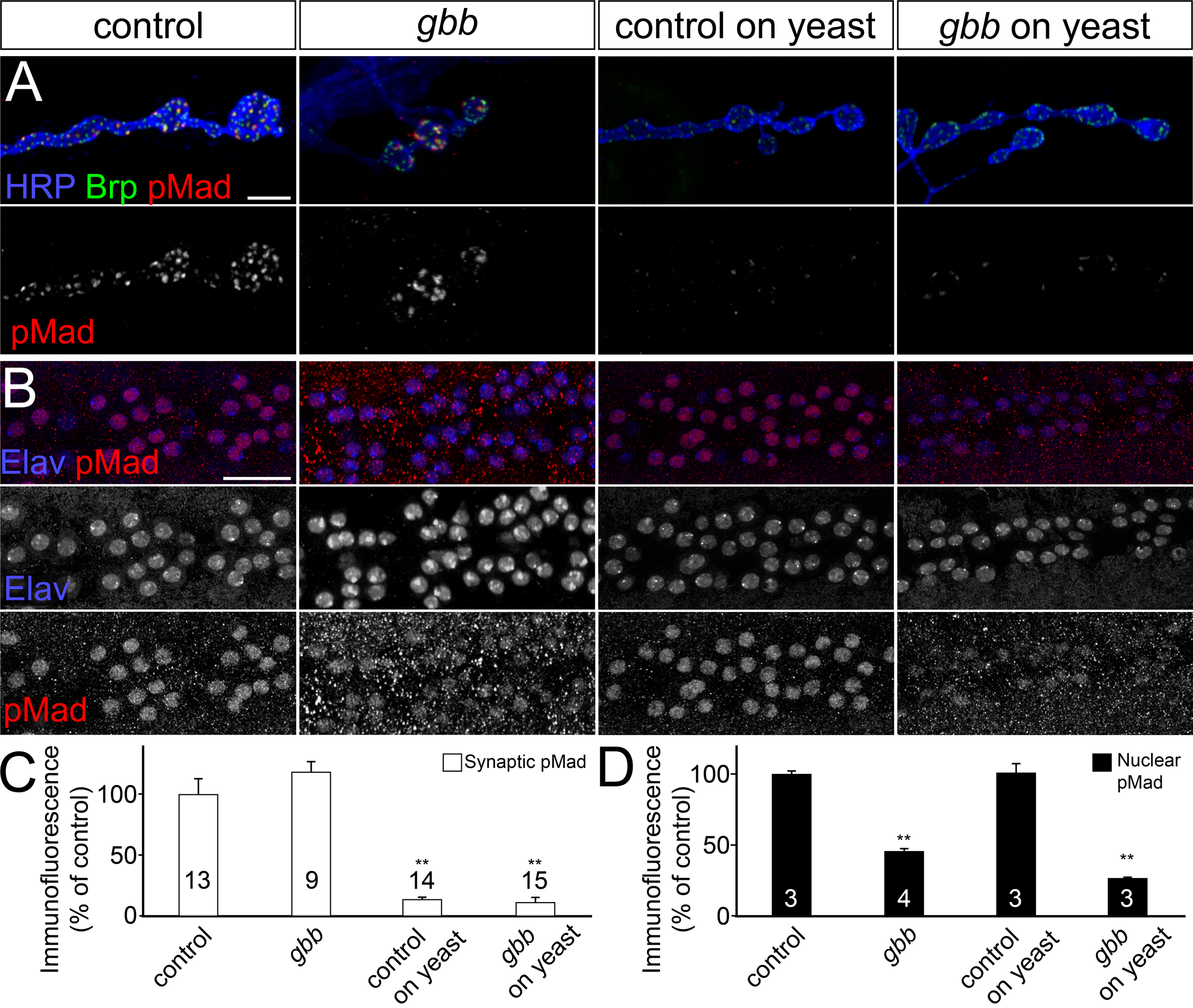

Supplement: S1 Fig — (A) (A-B) Confocal images of NMJ4 boutons (A) and ventral ganglia (B) from control and gbb mutant larvae immunolabeled for pMad (red), and Brp (green) and HRP (blue) (A) or Elav (blue) (B). Rearing larvae on a yeast diet largely eliminated synaptic pMad in both controls and gbb mutants. In contrast, nuclear pMad does not change. (C-D) Quantification of mean intensity for synaptic pMad (C) or nuclear pMad (D). Genotypes: control (w1118), gbb (gbb1/gbbDf). Error bars indicate SEM. **; p<0.001. Scale bars: 5 μm (A) and 20 μm (B). (TIF) [file pgen.1005810.s006.tif]

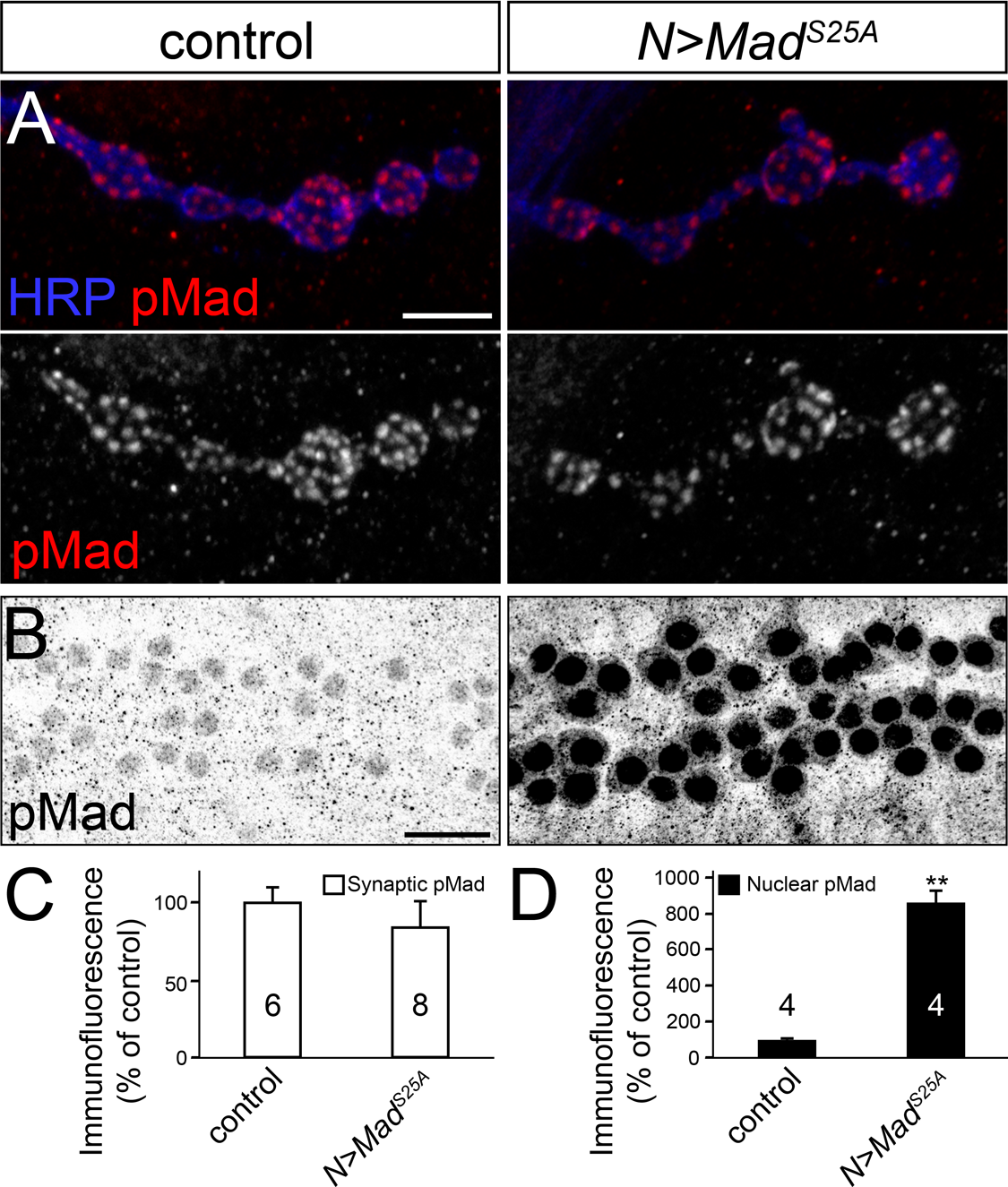

Supplement: S2 Fig — (A-B) Confocal images of NMJ4 boutons (A) and ventral ganglia (B) from control and third instar larvae with a phosphomutant (S25A) Mad variant overexpressed in motor neurons (N>MadS25A). Neuronal expression of MadS25A does not affect the synaptic pMad (A) while greatly increases the accumulation of nuclear pMad (B) (quantified in (C-D)). Genotypes: control (w1118), N>MadS25A (380-Gal4/Y; +; UAS-MadS25A/+). Error bars indicate SEM. **; p<0.001. Scale bars: 5 μm (A) or 15 μm (B). (TIF) [file pgen.1005810.s007.tif]

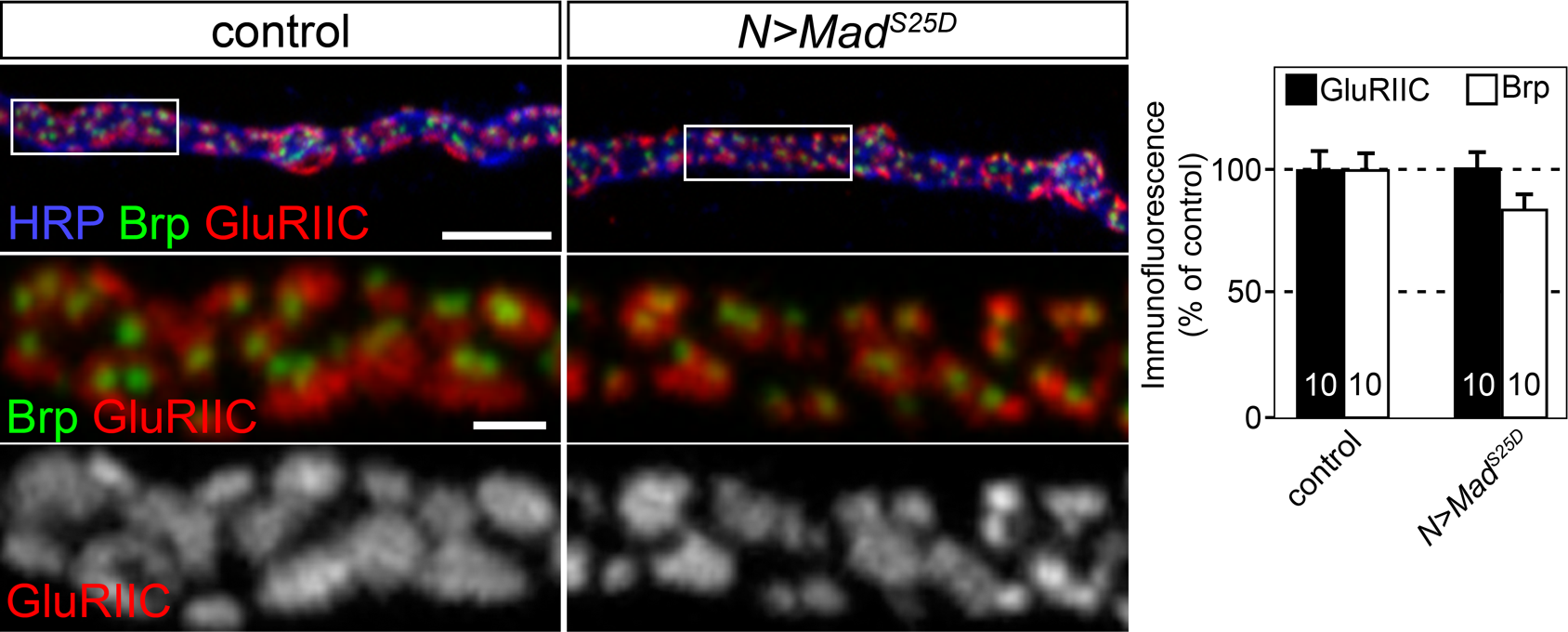

Supplement: S3 Fig — Confocal images of NMJ4 from third instar larvae labeled for HRP (blue), Brp (green), and GluRIIC (red). Excess presynaptic MadS25D does not alter the density and integrity of synaptic contacts as measured by juxtaposed Brp and GluRIIC signals. The net GluRIIC synaptic levels appear normal in animals with excess MadS25D compared to controls. Genotypes: control (380-Gal4/Y), N>MadS25D (380-Gal4/Y; +; UAS-MadS25D/+). Error bars represent SEM. Scale bars: 5 μm and 1 μm (details). (TIF) [file pgen.1005810.s008.tif]

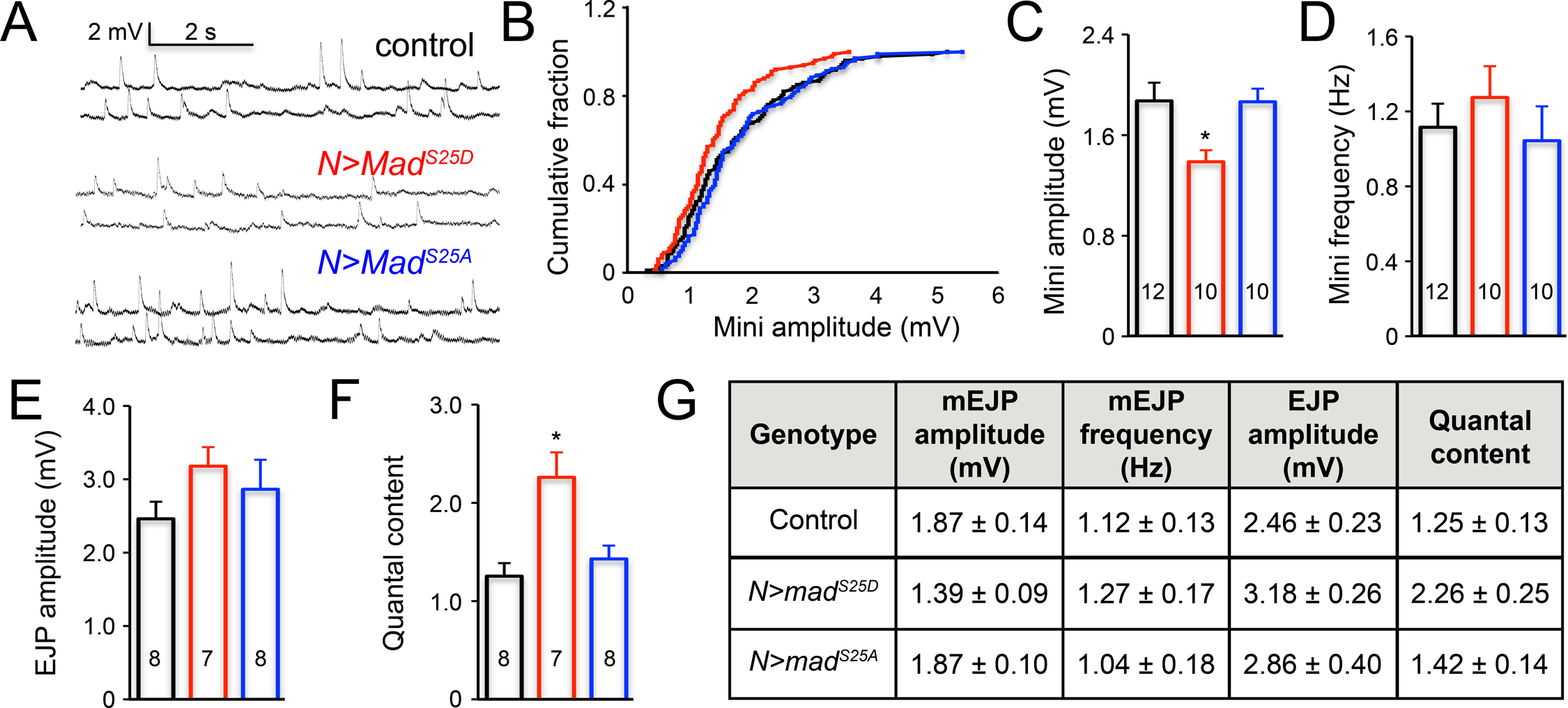

Supplement: S4 Fig — (A-G) Electrophysiological recordings from muscle 6, segment A3, of control and third instar larvae with excess presynaptic MadS25D (N>MadS25D) or MadS25A (N>MadS25A). Representative traces of mEJPs are shown in (A) and the results are summarized in (G). The number of NMJs examined is indicated in each bar. The mEJPs amplitude (B-C) but not frequency (D) was reduced when MadS25D was overexpressed in the motor neurons. However, the EJP amplitude was normal due to a significant increased in quantal content (E-F). The muscle resting potential and the input resistance were not affected. Genotypes: control (380-Gal4/Y), N>MadS25D (380-Gal4/Y; +; UAS-MadS25D/+), N>MadS25A (380-Gal4/Y; +; UAS-MadS25A/+). Error bars indicate SEM. *; p<0.01. (TIF) [file pgen.1005810.s009.tif]

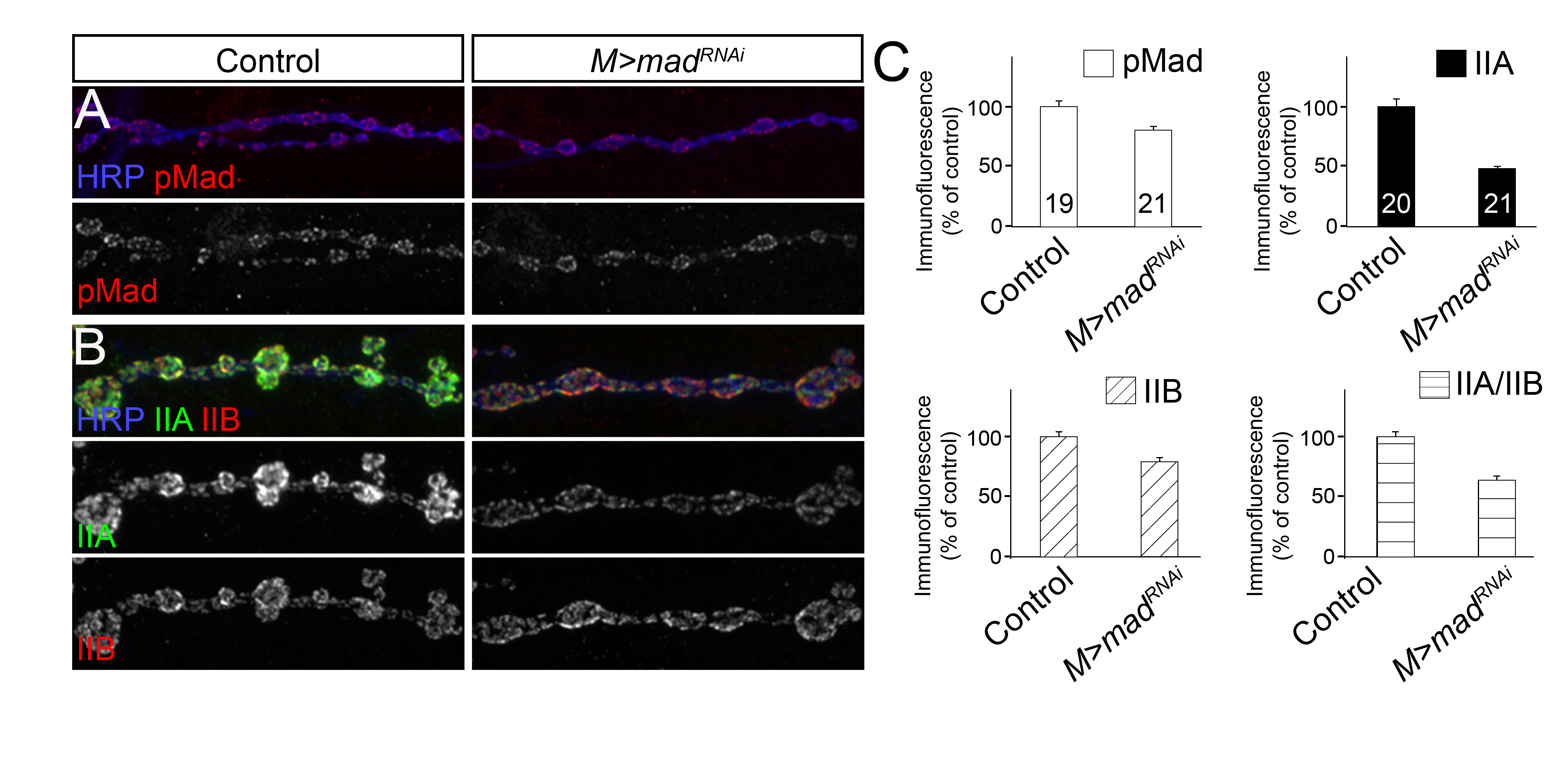

Supplement: S5 Fig — (A-B) Confocal images of NMJ4 boutons from larvae of indicated genotypes labeled for GluRIIA (green), pMad or GluRIIB (red) and HRP (blue). Mad-depleted muscles have mildly reduced synaptic pMad but severely disrupted GluRIIA synaptic levels (quantified in C). Genotypes: control (UAS-MadRNAi/+), M>MadRNAi (24B-Gal4/UAS-MadRNAi). Error bars indicate SEM. ***; p<0.001. (TIF) [file pgen.1005810.s010.tif]
